# Experimental and machine learning-based comparison of swirling and conventional conical fluidized bed reactors for enhanced thermal performance

**DOI:** 10.1038/s41598-026-48623-y

**Published:** 2026-04-24

**Authors:** Hamada Mohamed Abdelmotalib, Ahmed A. Abdel Samee, Mohamed Hamam M. Tawfik

**Affiliations:** 1 Department of Mechanical Power and Energy Engineering, Faculty of Engineering, Minia University, Minia, 61111 Egypt; 2 Department of Mechanical Engineering, Faculty of Engineering, Qena University, Qena, 83511 Egypt

**Keywords:** Bed hydrodynamic, Conical fluidized bed, Heat transfer coefficient, Machine learning, Swirling flow, Energy science and technology, Engineering, Mathematics and computing

## Abstract

The current study presents an integrated methodology for improving the performance of conventional fluidized bed reactors using swirling flow via a blade distributor and conical shape. The primary objective of this study is to investigate the combined effect of swirling flow and conical geometry on bed flow and heat transfer, which has not been sufficiently studied, especially through experimental techniques supported by machine learning modeling able to predict performance. A blade distributor was employed in the SCFBR, while the bed heat transfer coefficient and surface particle temperature were estimated and measured at different inlet velocities ranging from 0.993 to 2.5 m/s. The study results emphasized that the SCFBR clearly outperforms the CFBR based on lower bed pressure drop, more uniform particle distribution, and higher heat transfer coefficient. The heat transfer coefficient of the SCFBR increased by 40% compared to that of the CFBR, corresponding to a more homogenized distribution of particle surface temperature. The machine learning findings elucidate the superiority of the Extra Trees model in modeling the bed pressure drop and heat transfer coefficient, achieving with an R² of 0.973, an RMSE of 52.11, and a MAE of 35.11 for the heat transfer coefficient and an R² of 0.965, an RMSE of 8.76, and a MAE of 2.27 for the bed pressure drop. The good agreement between the experimental and ML results demonstrated the reliability of the proposed methodology. This study emphasizes the importance of integrating swirling flow with conical geometry, supported ML modeling, and represents a promising method for developing thermally efficient fluidized bed reactors with low energy consumption for advanced industrial applications.

## Introduction

Biomass is an abundantly available source of energy found in numerous types of organic feedstock, such as agricultural and forest wastes, as well as municipal solid wastes, which are used to generate energy. Compared to fossil fuels, bioenergy produced from different types of biomass can decrease CO_2_ and other greenhouse gases in the long term^1–3^. In spite of the fact that the majority of biomass resources are used for heating and cooking in rural and remote regions of developing countries, hopeful progress has been made in using biomass in thermal conversion processes such as combustion, gasification, and pyrolysis^4^. Fluidized bed reactors are an efficient technique used in converting biomass and other solid wastes into energy using different processes, including combustion, pyrolysis, and gasification. This is due to their numerous advantages, such as fuel flexibility, intensive mass and heat transfer, the ability for emission control, and excellent gas-solid mixing^5–7^. Conical (sometimes called tapered or divergent) and swirling fluidized beds are two types of fluidized beds that have been given more attention in recent studies. In spite of FBRs with a cylindrical shape are common, reactors with conical-bottoms are increasingly favorable in thermochemical processes of biomass, such as gasification and pyrolysis. The conical geometry can handle a wide range of particle sizes and decrease stagnant zones by generating a high-velocity region at the distributor level that gradually decelerates, which decreases the entrainment of particles. The conical FBRs also exhibit several other advantages compared to cylindrical reactors, such as a small quantity of bed material, lower bed pressure drop, shorter start-up time, decreased gas consumption, and the ability to use particles with different properties^8–10^. Therefore, conical FBRs have been used in a wide range of industrial applications, such as catalytic polymerization, waste material incineration, fluidization of cohesive powders, wastewater biological treatment, biomass processing, drying, and in the chemical and petroleum industries^11–18^. In order to enhance the performance of the fluidized bed reactor and overcome some problems like channeling and slugging, a swirling fluidized bed (SFB) is used. In this type, the axial motion of the fluidized agent is replaced by swirling motion^19^. The swirling motion can be created via blades or nozzles, which results in minimizing the axial momentum that is converted into radial and tangential momentum^20^. Swirling fluidized bed reactors have numerous advantages compared to conventional types, such as lower bed and distributor pressure drop, higher gas velocity with lower elutriation of small particles, the capability of using large bed particles, better heat and mass transfer, and good mixing in the radial direction^21,22^.

Regardless of the advantages of both conical and swirling fluidized bed reactors, there are few studies focused on the heat transfer process and bed flow behavior in these reactors compared to conventional types. Das et al.^8^ studied the heat transfer in conical FBR at different inlet air velocities and a cone angle of 10° using 3D numerical simulations and experiments. The results indicated that the lowest pressure drop and the highest bed temperature were achieved at a velocity of 2 m/s, which is considered the best inlet velocity. At the central axial locations of 10 and 20 cm, the bed-wall heat transfer coefficient increased by 47.2% and 45.8%, respectively. Zhang et al.^23^ numerically studied the bed-wall heat transfer using pulsed flow. There was a strong relation between heat transfer and bed hydrodynamics. The bed-surface heat transfer coefficient improved by decreasing the taper angle and decreasing the distance between the gas inlet and probe location. The results also indicated that the heat transfer crumbled by using rectangular pulsed flow with a long-time off duration. Abdelmotalib et al.^24^ investigated the heat transfer between an immersed heater and a cone bed using a 3D model of a conical FBR and an Eulerian–Eulerian model. The heat transfer and bed hydrodynamics were studied using different inlet velocities and different particle-wall boundary conditions. Increasing inlet air velocity increased the bed expansion ratio and enhanced the heat transfer process. The best agreement between numerical and experimental results was achieved by applying no-slip conditions. In other studies, by Abdelmotalib et al.^25,26^, the effects of the specularity coefficient, particle-to-particle and particle-to-wall coefficients of restitution on heat transfer and bed flow were studied. The results demonstrated that the wall-bed heat transfer coefficient decreased with increasing specularity coefficient, while this increase didn’t affect bed pressure drop. The bed pressure drop increased with increasing particle-to-particle restitution coefficient, while it decreased with increasing the particle-to-wall coefficient of restitution. Both the particle-to-particle restitution coefficient and the particle-to-wall coefficient of restitution had a small influence on the heat transfer process.

Like conical FBR, there are few studies about heat transfer and hydrodynamics in swirling FBR. Wu et al.^27^ selected a hybrid Eulerian-Lagrangian model to study the heat transfer process in a swirling fluidized bed. The bed-wall and gas-solid heat transfer processes, temperature fields, and solid hold up are predicted. The results indicated that increasing operating velocity improved the mixing and heat transfer processes. Also, decreasing the inclination angle from 45° to 12° increased the bed-wall heat transfer coefficient by 20%. In another study by Wu et al.^28^, a Dense Discrete Phase method was used to study bed hydrodynamics, the behavior of particle mixing, and wall wear in a swirling FBR with an air plenum. Increasing superficial velocity led to the development of different operational regimes. In the stable swirling regime, the bed particles were subjected to strong elutriation and were centrifugally attached to the reactor wall. Both swirling velocity and bed pressure drop decreased with the reduction of central body height. The influence of operating parameters and reactor design on the heat transfer coefficient in a swirling twin-cyclonic FBR was studied by Sirisomboon and Laowthong^29^. Both secondary and tertiary air were used with different ratios ranging from 0 to 0.5 at primary air velocities ranging from u_mf_ to 3u_mf_, using sand with different sizes as bed material. The heat transfer radial profiles were maximum at the reactor center. The heat transfer coefficient increased with increasing air distributor swirl number and the flow rate of the primary air. Using experimental results enabled the development of semi-empirical models to predict average Nusselt numbers in freeboard and dense regions. The developed models were in a good agreement with experimental results within an error of ± 20% for the two regions. Tawfik et al.^30^ experimentally studied the heat transfer in a swirling FBR with a blade distributor at different operating parameters. The experiments were conducted in a swirling FBR with a length of 50 cm and a diameter of 10 cm using sand particles with a mean diameter of 1.5 mm. The results demonstrated that the heat transfer coefficient increased with an increasing number of distributor blades and decreased with the use of an air plenum and central bodies. The distributor and bed pressure drop decreased with increasing number of the distributor blades and the use of gas plenum, and increased with using central bodies. The effect of using a binary mixture composed of sand and polyethylene (PE) on heat transfer and bed flow behavior was investigated in another study by Tawfik et al.^31^. The bed-wall heat transfer coefficient and pressure drop were calculated at different inlet velocities and different mass fractions of 0.25, 0.5, and 0.75 of PE beads. Increasing the mass fraction of PE beads resulted in increasing minimum fluidizing and swirling velocities, increasing bed pressure drop, and decreasing heat transfer coefficient. Using a central body led to a reduction in the bed pressure drop and an increase the heat transfer coefficient.

Although both conical and swirling fluidized bed reactors have many advantages, a critical study of the current literature elucidates various significant gaps. The majority of studies have separately considered either the effect of swirling flow or conical geometry, without considering their combined influence on bed hydrodynamics and heat transfer processes. Additionally, while numerical simulations have offered insight into bed hydrodynamics, they often lack strong experimental validation using non-intrusive and high-resolution measurement methods such as infrared thermography. Several previous studies also depend on semi-empirical correlations that may not accurately capture the complex nonlinear interactions between operating variables. There is a significant requirement for a hybrid approach that integrates experimental findings with advanced predictive modeling, such as ML models, to enable a more comprehensive and reliable tool to optimize the performance of FB reactors. To address these gaps, the novelty of this study is the integration of swirling flow, using blade distributor, in a conventional conical fluidized bed reactor and studying the impact of this integration on hydrodynamic behavior and heat transfer. This study is distinguished by its use of infrared thermography to measure the non-interference particle temperature distribution, in addition to the development of ML model able to predict heat transfer coefficient and pressure drop. The key objectives of this study are to (i) estimate and compare the bed flow parameters, mainly minimum fluidizing and swirling velocities and distributor and bed pressure drops for two distributor configurations; (ii) evaluate the bed-to-wall heat transfer coefficient and surface particle temperature distribution at different inlet velocities; and (iii) achieve ML modeling to complete the experimental results, addressing some parameters that are difficult to capture experimentally and providing a reliable predictive framework. To perform these objectives, an integrated approach combining experiments and artificial intelligence models was adopted. The experimental work aims to measure pressure drop and heat transfer coefficient at different inlet velocities and measure surface particle temperature using an infrared thermography. The machine learning models, including Extra Trees, decision tree regression (RT), and support vector regression (SVR), were developed to predict both the heat transfer coefficient and pressure drop based on operational variables. This framework provides comprehensive insight into the heat transfer and hydrodynamic performance of the FBR and intensifies its potential for development and application in advanced industrial systems.

## Material and method

### Materials

In this study, sand particles were used as bed material. Sand was mostly used as bed material because of its availability, low cost, and good thermal properties, while air was used as the fluidizing gas. Table 1 gives the thermophysical properties of sand particles and air, taken at standard ambient temperature of 25 °C. In addition to the properties provided in the table, other properties of the bed material were estimated, such as sphericity (0.9) which affects the drag coefficient and internal mixing, and volume fraction (0.4), which determines the start of fluidization and gas distribution in the bed.

**Table 1:** Thermophysical properties of sand and air used in this study.

Property	Sand particles	Air
Mean diameter (mm)	1.8	-
True density (kg/m^3^)	2600	1.2
Bulk density (kg/m^3^)	1450	1.2
Specific heat (J/kg K)	800	1005
Thermal conductivity (W/m K)	0.2	0.025

### Experimental test rig

**Fig. 1:**
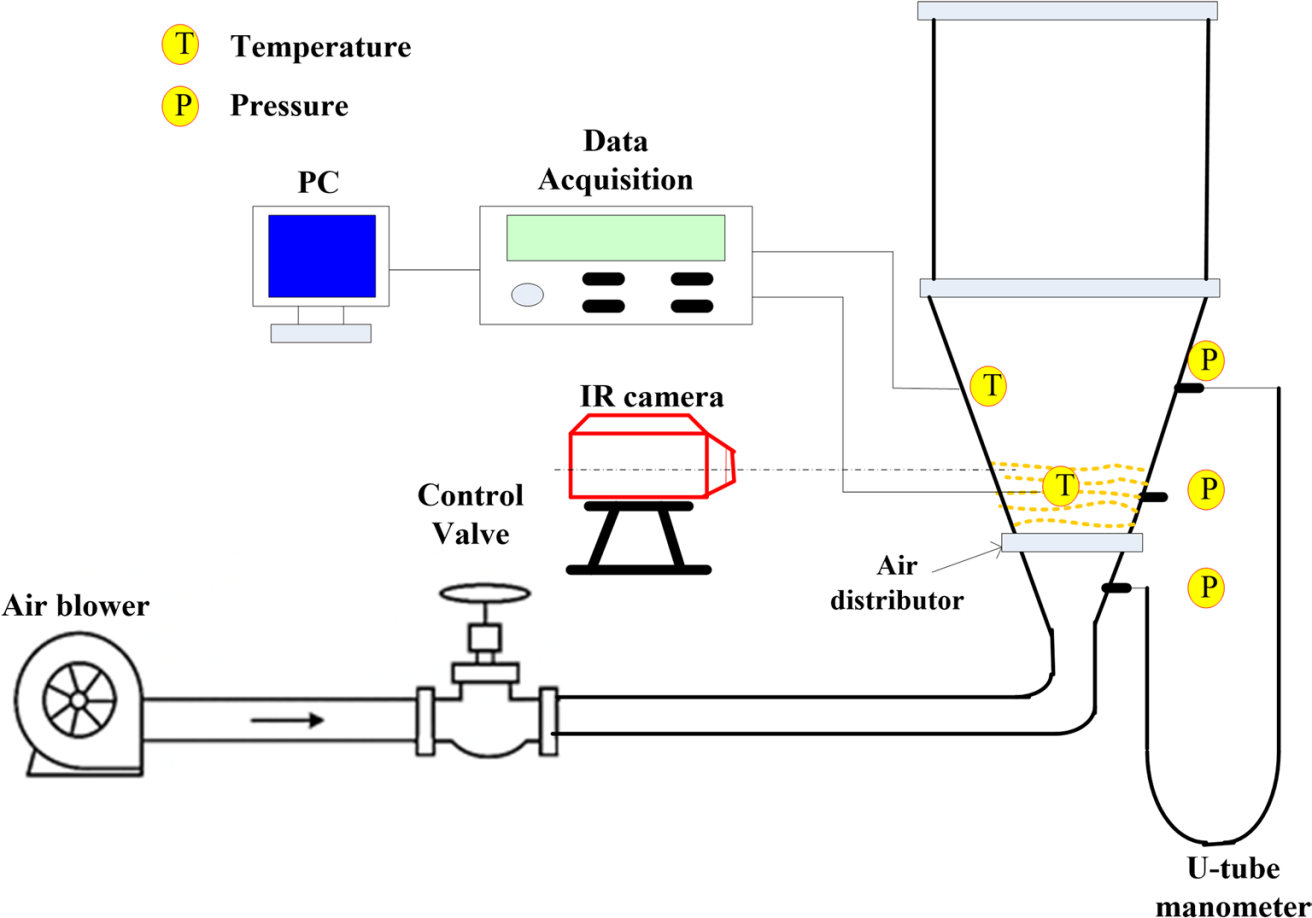
The schematic diagram of the experimental test rig used in this study.

**Fig. 2:**
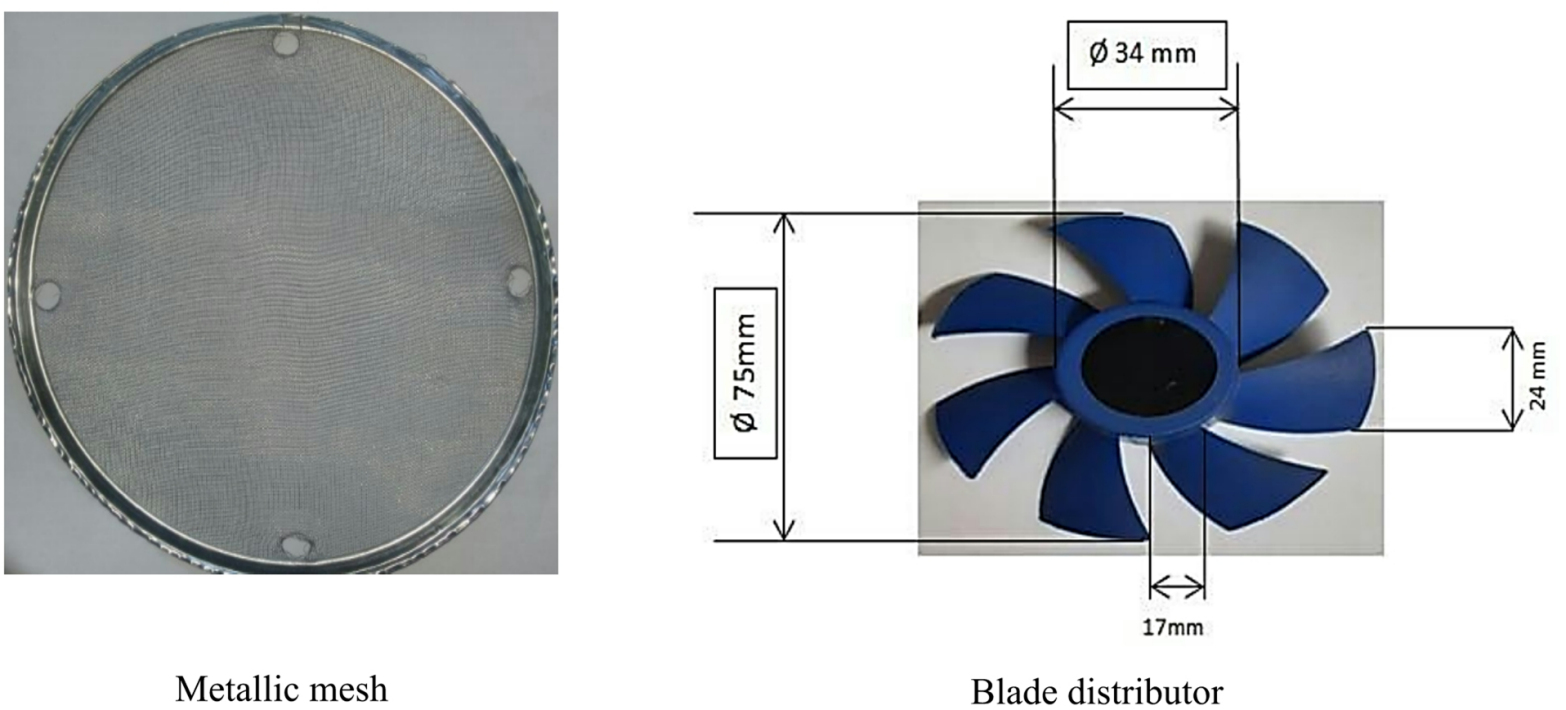
Photos of conventional and swirling air distributors used in the experiments.

The test rig used in this study is illustrated in Fig. 1. The test rig is composed of a fluidized bed reactor and measuring devices. The fluidized bed reactor is composed of two parts; the lower part is conical with inlet and outlet diameters of 9 cm and 14 cm, respectively and a total length of 30 cm. The upper part has a square shape with a width of 14 cm and a height of 20 cm. The reactor is made from steel with a thickness of 2 mm. The geometry of the reactor changes from a conical shape in the lower part to a rectangular shape in the upper part. This specific transition of the geometry was adopted to enable and facilitate accurate experimental measurements. The rectangular shape with flat surfaces in the freeboard region minimizes the optical distortion of curved surfaces, thereby achieving accurate measurements using infrared thermography. It also allows for more secure mounting and is easier to attach thermocouples and pressure taps, which ensures a flush fit with the internal wall to prevent flow disturbances. The rectangular shape is used in many large boilers and industrial heat exchangers for easier arrangement of internal tube bundles. There is a Perspex window in the lower part of the reactor for visual observations and measuring the particles surface temperature. Air is fed into the system using an air blower and passes through a control flywheel valve with an inner diameter of 7.5 cm, while its velocity is measured using a hotwire anemometer (Tenmars-402) with a range of 0.4–20 m/s, a resolution of 0.1, and an accuracy of (± 0.2%). The bed and distributor pressure drops are measured using a U-tube manometer connected to pressure taps. The temperatures along the axial and radial directions are measured using thermocouples type K connected to a data acquisition system (Tenmars-82n) with a range of -200 °C to 1370 °C, a resolution of 0.1, and an accuracy of ± 0.05% reading + 0.7 °C. The surface particle temperature was measured using an infrared camera (UNI-T-UTi712S), a cost-effective thermal camera with a range of -20 °C to 400 °C, a resolution of 0.1 °C, and an accuracy of ± 2 °C or ± 2%. The inlet air is heated using two tubular heaters, each with a total power of 1000 W, and the inlet air temperature is controlled by adjusting the inlet voltage via a variable voltage device (variable transformer, GB7676-87) with a range of 0 to 300 V and a step of 5 V. In the experiments sand with a diameter of 1.8 mm and a static height of 7 cm was used as bed material. In this study, two types of air distributors, as shown in Fig. 2, were used. The first one is a metallic mesh with a hole size of 0.12 mm, representing the conventional CFBR. The other distributor is an annular-blade distributor used to generate swirling motion. The blade distributor is composed of 7 blades, and they are located at an angle of 45° to the horizontal; it is covered with the same mesh to prevent sand particles from falling down.

### Experimental method

In this study, the bed flow characteristics and heat transfer process are analyzed. The bed flow behavior is analyzed using different parameters, including minimum fluidizing and swirling velocities, as well as bed and distributor pressure drop. The heat transfer process is analyzed by measuring parameters such as bed and surface temperature, surface particle temperature, and calculating the bed-to-wall heat transfer coefficient. The minimum fluidizing velocity (used in conventional FBR), at which the bed starts to fluidize and the minimum swirling velocity (used in swirling FBR), at which the bed particles start to swirl are measured using visual observation and compared to those calculated from correlations. The distributor pressure drop is measured using two pressure taps located at a distance of 1 cm before and after the air distributor. The bed pressure drop is measured using two pressure taps located at the inlet and outlet of the reactor.

The bed-to-wall heat transfer coefficient is calculated by measuring both bed and surface temperatures at different axial and radial positions using the following equations:

1 $$\:h=\frac{Q}{{A}_{s}({T}_{b}-{T}_{S})}=\frac{\dot{m}\:{C}_{p}\varDelta\:{T}_{a}}{{A}_{s}({T}_{b}-{T}_{s})}$$

Where Q is total input heat, $\:{\dot{m}}\:$is the input air mass flow rate (kg/s), *C*_*p*_ is the air specific heat capacity, $\:\varDelta\:{T}_{a}$ is the temperature difference between the measured air temperature and atmospheric temperature. $\:{A}_{s}$ surface bed area at the measuring location. *T*_*b*_ and *T*_*s*_ are the measured bed and surface temperatures, respectively. The experiments were carried out three times and the averaged values are used in the calculations.

### Infrared thermography measurement and calibration

The particles’ surface temperature was measured using an infrared camera (UNI-T-UTi712S) positioned perpendicular to the plexiglass window, with a thickness of 3 mm and a transmittance of 0.87, of the reactor, at a distance of 0.5 m. The camera was calibrated with an emissivity value of 0.92, suitable for silica sand in the infrared range (8–12 μm), and the ambient temperature (25 °C) and the transmittance of the plexiglass wall were also taken into account. A thermocouple type K (1 mm diameter, accuracy ± 0.5 °C) placed close to the inner wall was used to calibrate the camera. The results of calibration process indicated a linear relationship between the camera and the thermocouple readings, with a slope of 0.98 and an offset of + 1.6 °C (R² = 0.993). These calibration parameters were used to correct all the recorded thermal readings. The spatial resolution of the thermal images was 0.7 mm per pixel at the measurement distance, while the temporal resolution was 30 frames/second. To minimize reflections and background radiation, the surroundings were covered with a non-reflective (matte black) material, and the measurements were taken under stable lighting conditions. The effective measurement uncertainty of the camera after correction (based on device specifications, transmittance correction, and linear regression residuals) was estimated to be approximately ± 1.5 °C at a 95% confidence level. Therefore, the surface temperature values ​​of the particles presented in this study have been corrected for emissivity and glass window transmittance and verified through calibration. The average temperature values ​​were calculated by averaging 300 consecutive frames after reaching a steady state, in order to minimize measurement variability.

### Uncertainty analysis

The uncertainty analysis is conducted to achieve the reproducibility and reliability of the experimental measurements using the Kline and McClintock method^32^, which presents a systematic approach to estimate the propagation of uncertainties in derived experimental variables. This approach is suitable when the experimental parameter relies on multiple measured variables, each with its own uncertainty.

The uncertainty of the heat transfer coefficient can be determined using the following equation:

2 $$\:\frac{{W}_{h}}{h}={\left[{\left(\frac{{w}_{Q}}{Q}\right)}^{2}+{{\left(\frac{{w}_{\varDelta\:T}}{\varDelta\:T}\right)}^{2}+\left(\frac{{w}_{{A}_{s}}}{{A}_{s}}\right)}^{2}\right]}^{0.5}$$

Where *Q* is the input heat, *ΔT* is the temperature difference between the bed and walls, and *A*_*s*_ is the heat transfer surface area. Taking the uncertainty in heat input, temperature difference, and surface area as 0.02 W, ± 1.5 °C, and 0.005m^2^, the uncertainty in the heat transfer coefficient was calculated to be ± 2.8% W/m^2^.

The distributor and bed pressure drop were measured using a digital manometer in mm of water. The uncertainty of the pressure drop was calculated using the following equation:

3 $$\:\varDelta\:P=\rho\:g\varDelta\:{H}_{manometer}$$

4 $$\:\frac{\partial\:(\varDelta\:P)}{\varDelta\:P}={\left[{\left(\frac{\left(\frac{\partial\:\left(\varDelta\:P\right)}{\partial\:(\varDelta\:H)}\right)}{\varDelta\:P}{w}_{H}\right)}^{2}\right]}^{0.5}=\frac{{w}_{H}}{\varDelta\:H}$$

The uncertainty in pressure drop only depends on the uncertainty of the liquid height of the manometer ($\:{w}_{H}$*)*, therefore the uncertainty in pressure drop for the distributor and bed was determined to be 5%.

## Machine learning model

**Fig. 3:**
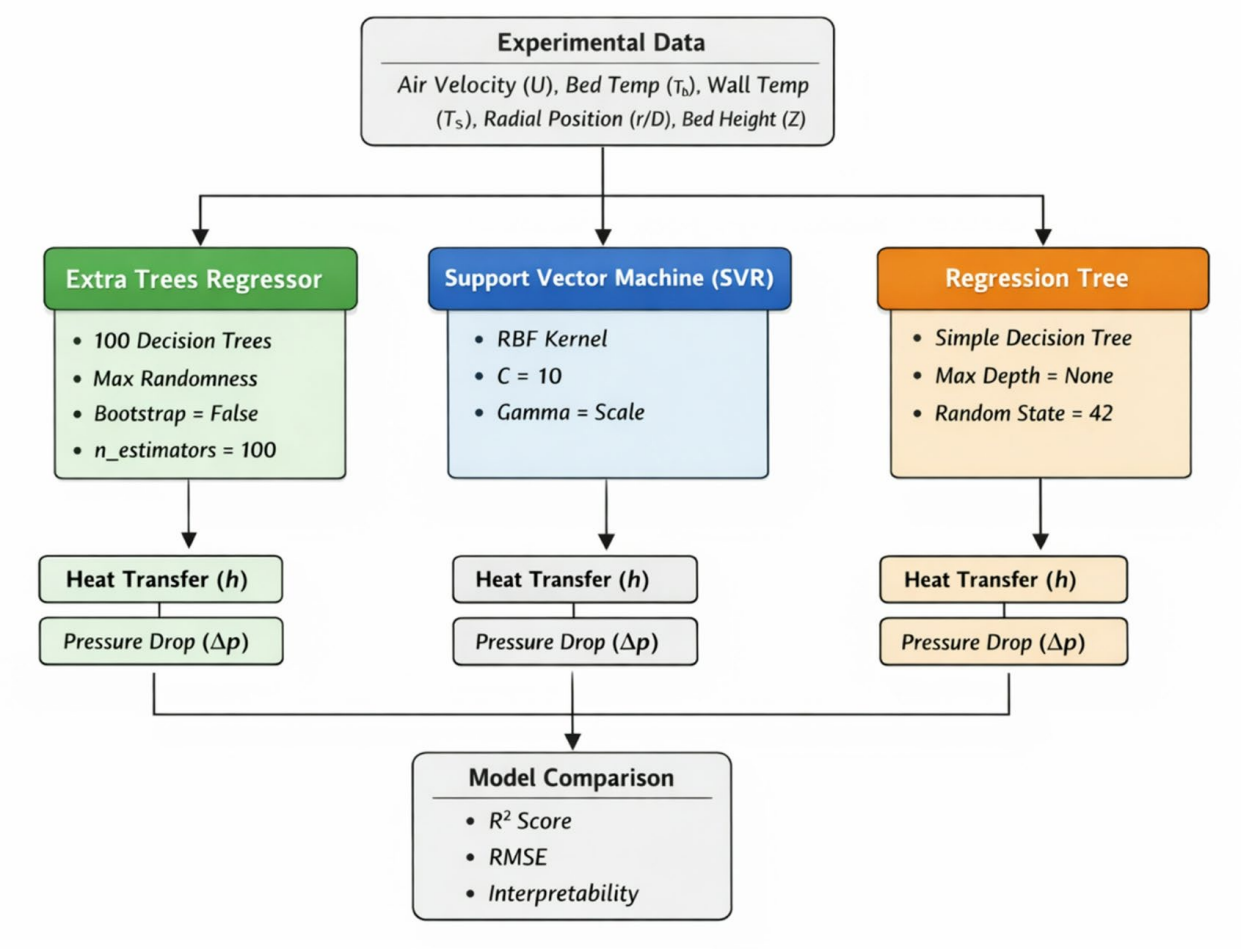
The proceduer of ML models development.

**Fig. 4:**
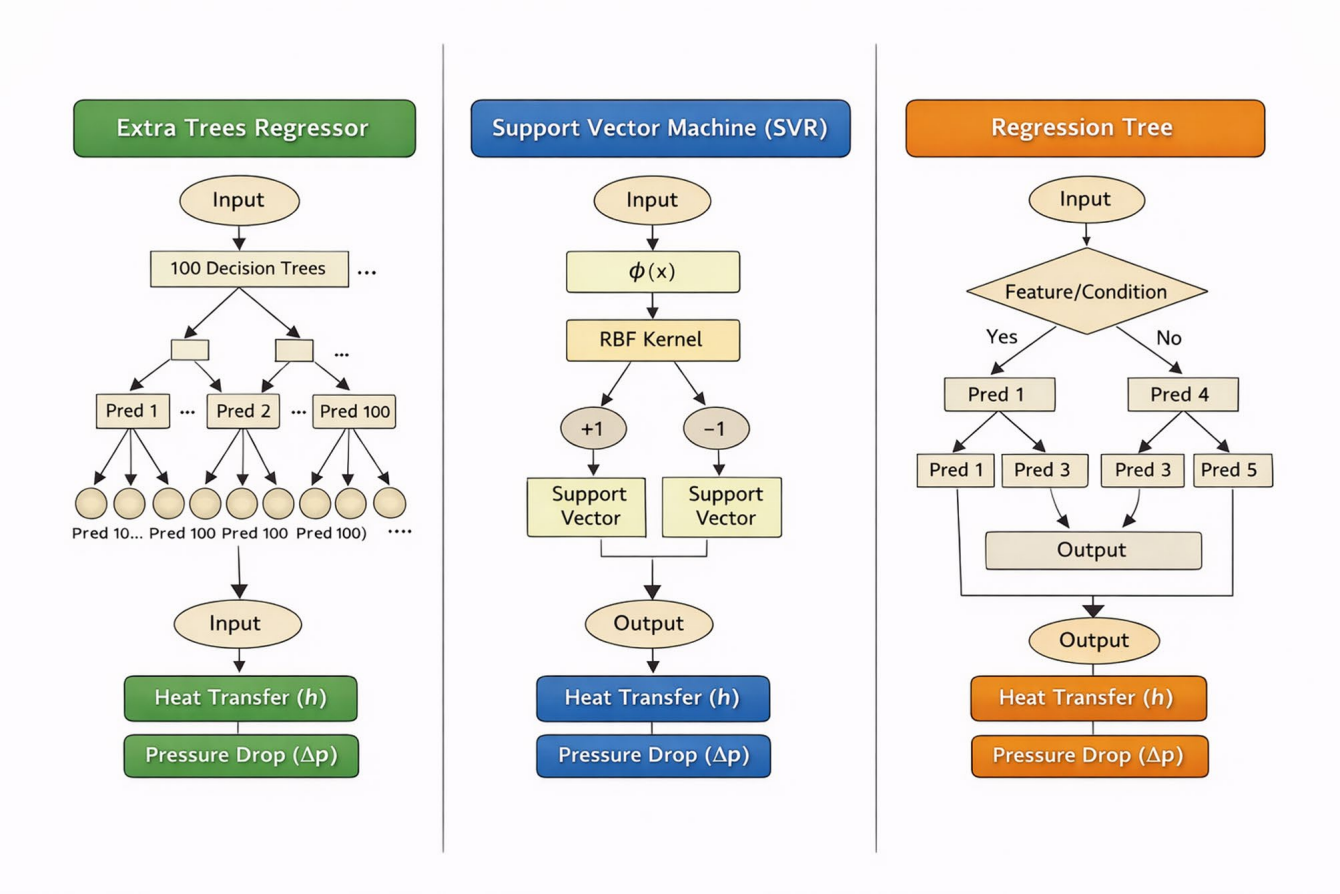
The structure of ML models used in this study.

In this study, three predictive ML models, as shown in Fig. 3, were developed to model heat transfer and bed pressure drop in SCFBR and CFBR. The input parameters of these models were the experimental results, including air inlet velocity (U = 0.933–2.49 m/s), bed temperature (Tb), wall temperature (Ts), relative radial position (r/D), and bed height (Z), while the target outputs are the heat transfer coefficient (h) and pressure differential across the bed (ΔP). The developed models are Extra Trees Regressor (ETR), Support Vector Machine (SVR), and Regression Tree (RT). Each model was trained on a dataset containing 64 datasets representing the heat transfer coefficient results (8 points for each of 8 experimental cases) and 8 pressure drop points. Despite the small size of the dataset, which involves 64 high-fidelity experimental results for the bed pressure drop and heat transfer coefficient, it provides a detailed mapping of the operation of the reactor under controlled steady-state conditions. Based on experimental thermal sciences, data points are governed by underlying physical laws; such a size of dataset is sufficient for ensemble learning methods. Among the selected models, the ETR model was specifically chosen because of its capability to reduce overfitting on limited datasets via an ensemble of 100 randomized decision trees. This model can capture the primary physical trends of the SCFBR with high accuracy in prediction. Figure 4 illustrates the structure of each model used in this study. For the ETR model, the maximum randomness was used for the partition thresholds selection with max_features of 1.0 and bootstrap set to False, and with randomness for the cutoff thresholds. This increases variance and decreases bias, which makes the model more effective at handling complex nonlinear relationships in FBR. To achieve a balance between computational complexity and accuracy, the n_estimators (number of trees) was set to 100. A kernel function was used in the SVR model as it has high suitability for nonlinear datasets. The gamma parameter was set to its default value to prevent overfitting, given the limited sample size, and the regularization parameter (C = 10) was set to improve margins while allowing for some soft margins. The RT model was used with randomness fixed (random_state = 42) to achieve reproducibility. This model offers a transparent interpretation of relationships and helps in understanding the effect of factors. The data were divided into 30% testing and 70% training, with the seed fixed (random_state = 42) to achieve comparison fairness. These three models were deliberately chosen to cover a wide range of learning methodologies: ensemble methods ETR, SVR, and RT, allowing for a reliable assessment of generalizability and the model’s ability to generalize beyond the training set, especially given the challenge of the small dataset size (32 samples after expansion to long format). The performance of models was evaluated using statistical performance metrics: coefficient of determination (R²), mean absolute error (MAE), and root mean squared error (RMSE).

## Results and discussion

### Hydrodynamic parameters for conventional CFBR and SCFBR

**Fig. 5:**
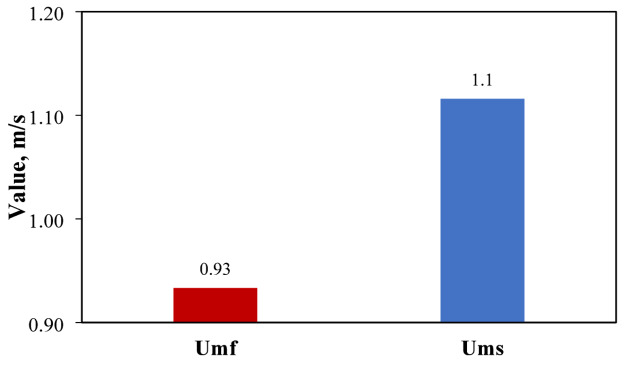
The minimum fluidizing and swirling velocities for CFBR and SCFBR.

The comparison between the minimum fluidizing and minimum swirling velocities is shown in Fig. 5. The minimum fluidizing velocity represents the minimum velocity at which fluidization of particles is observed in a conventional FBR. The minimum swirling velocity is the minimum velocity at which the swirling motion starts in SCFBR. The measured values of minimum fluidization and swirling velocities were 0.93 m/s and 1.1 m/s, respectively, indicating a significantly higher swirling velocity. The rise in minimum velocity reflects the additional energy needed to initiate the swirling motion in the bed because of the swirling effect of the blade distributor. Using a blade distributor generates swirl action that promotes the hydrodynamic behavior of the fluidizing medium, improving the mixing process and providing a high uniform distribution of particles. The calculated value of minimum swirling velocity suggests a higher dynamic flow region associated with enhanced mass and heat transfer processes, which are beneficial and advantageous for different thermochemical conversion processes achieved in these reactors.

**Fig. 6:**
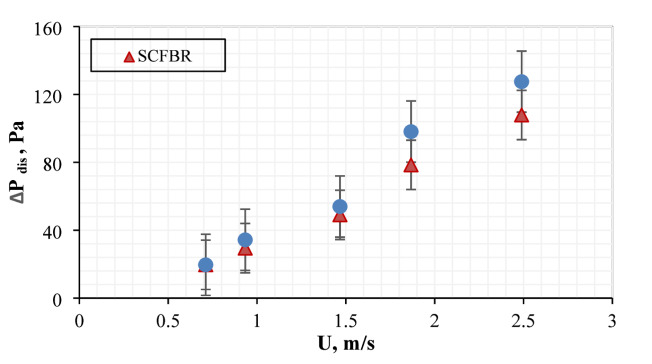
The distributor pressure drop for CFBR and SCFBR at different inlet velocities.

The variation of distributor pressure with inlet velocity for conventional CFBR and SCFBR is illustrated in Fig. 6. The distributor pressure drop increased with an increase in inlet velocity for both reactors, consistent with improved momentum transfer to the particles and flow resistance. The distributor pressure drop of conventional CFBR was higher than that of SCFBR at all inlet air velocities, elucidating that the blade distributor decreases the localized resistance at the level of the distributor and produces a more efficient distribution of gas. This behavior may be attributed to the rotational flow component, which improves the interaction between bed particles and fluidizing gas and prevents an increase in pressure accumulation in the bottom region. The decrease in distributor pressure drop in SCFBR reflects the savings in energy in the gas supply system, along with enhanced hydrodynamic stability, which are essential in reactor scale-up and continuous operation.

**Fig. 7:**
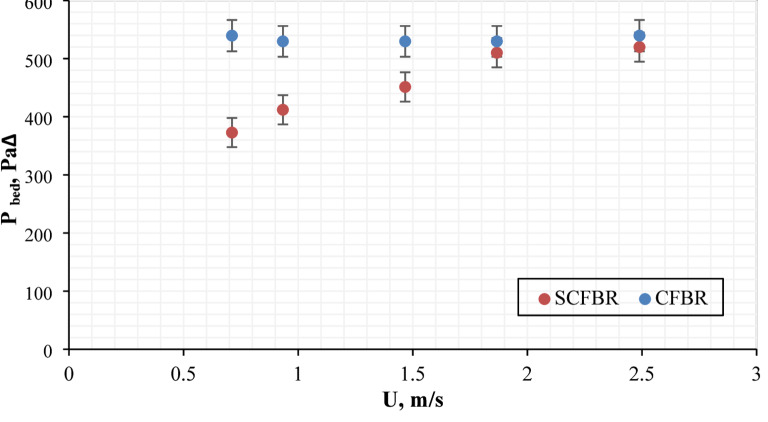
The bed pressure drop for CFBR and SCFBR at different inlet velocities.

Figure 7 illustrates the variation of bed pressure drop with inlet gas velocity for conventional CFBR and SCFBR. In both distributor configurations, the increase in inlet gas velocity increased the bed pressure drop, demonstrating the increase in drag force exerted by the gas on the bed particles. At all different inlet air velocities, SCFBR exhibited a lower bed pressure drop compared to CFBR. The reduction in bed pressure drop for SCFBR may be due to the swirling flow action produced by distributor blades, which decreases channeling, increases uniform fluidization, and promotes smoother circulation of bed particles. The swirling motion could decrease the resistance and localized packing in the bed, resulting in a decrease in the required pressure for keeping fluidization. The reduction in distributor and bed pressure drop for SCFBR confirms its improved hydrodynamic efficiency, distinguishing it as a hopeful design to enhance the use of energy with better control of gas-solid interaction in the operation of FBR. These results highlight the enhanced hydrodynamic efficiency of the SCFBR, making it a promising design for improved energy utilization and better control over fluid–solid interactions in industrial fluidized bed operations.

### Heat transfer for conventional CFBR and SCFBR

**Fig. 8:**
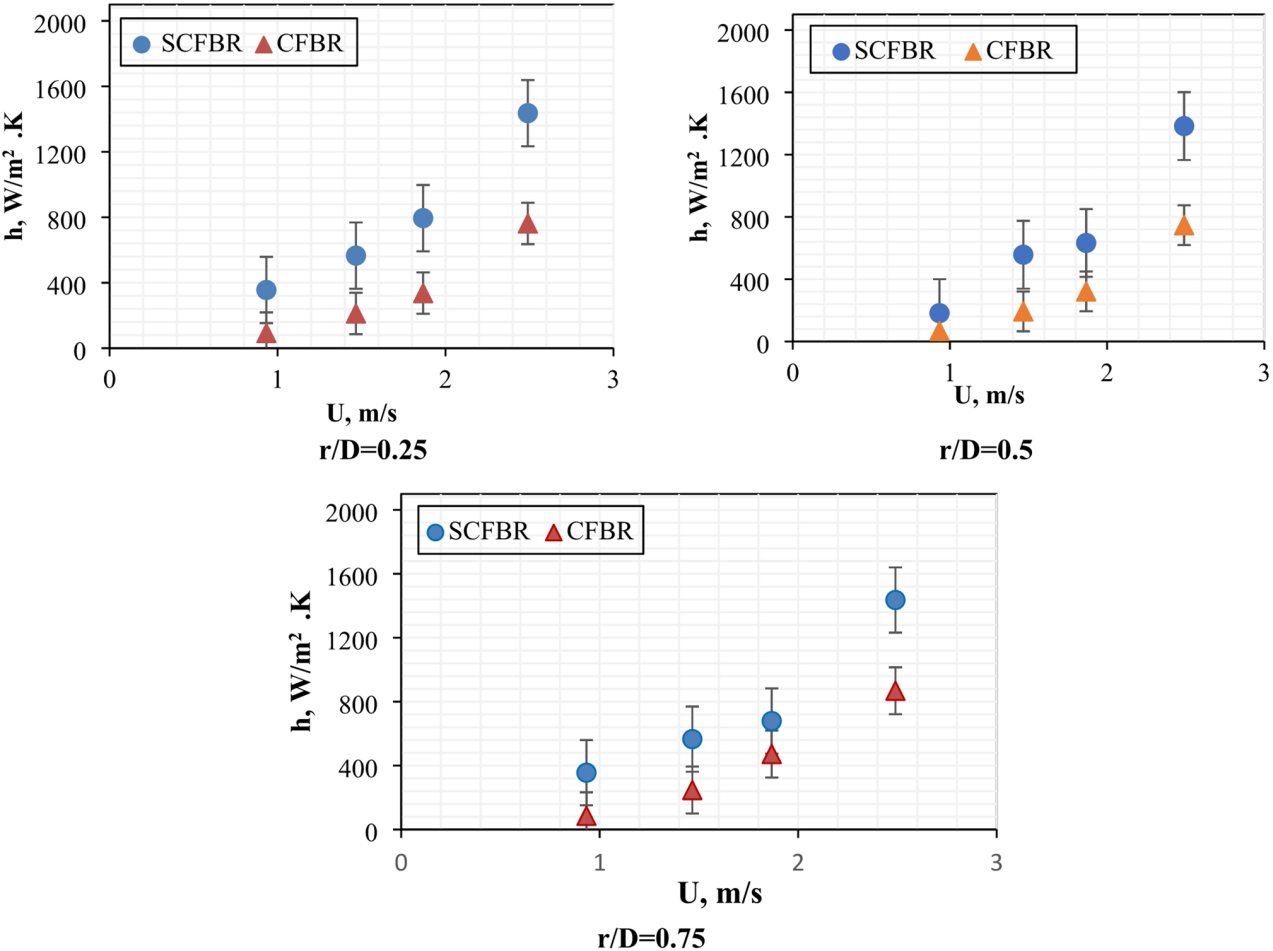
Radial distribution of heat transfer coefficient for CFBR and SCFBR at different inlet velocities, radial positions, and Z = 2.5 cm.

The radial distribution of the bed-to-wall heat transfer coefficient (h) for both conventional CFBR and SCFBR at different inlet velocities and a fixed bed height (Z) of 2.5 cm is shown in Fig. 8. The heat transfer coefficient was calculated at three radial positions normalized by the diameter of the reactor (r/D = 0.25, 0.5, and 0.75). As the figure indicates, the SCFBR had a higher heat transfer coefficient at all radial positions compared to the CFBR, indicating a significant difference as the gas velocity increased. At the radial position of r/D = 0.25, the heat transfer in the SCFBR improved due to the high interaction between the solid particles and reactor walls, as well as the intense localized mixing, enhanced by the swirling motion at the reactor center. At r/D = 0.5, representing the center of the reactor, the effect of swirling motion remains evident, suggesting particles’ uniform distribution and enhanced bulk flow dynamics. At r/D = 0.75, near the reactor wall, the highest improvement in heat transfer is achieved due to the significant rise in the tangential velocity of particles caused by swirling flow, which improves the strong contact of particles with the wall and increases convective and conductive heat transfer mechanisms. Overall, the obtained results confirm the ability of the SCFBR to improve the magnitude and uniformity of the heat transfer process by producing swirling motion, disrupting the dominance of axial flow, and considering the multidirectional particle movement.

**Fig. 9:**
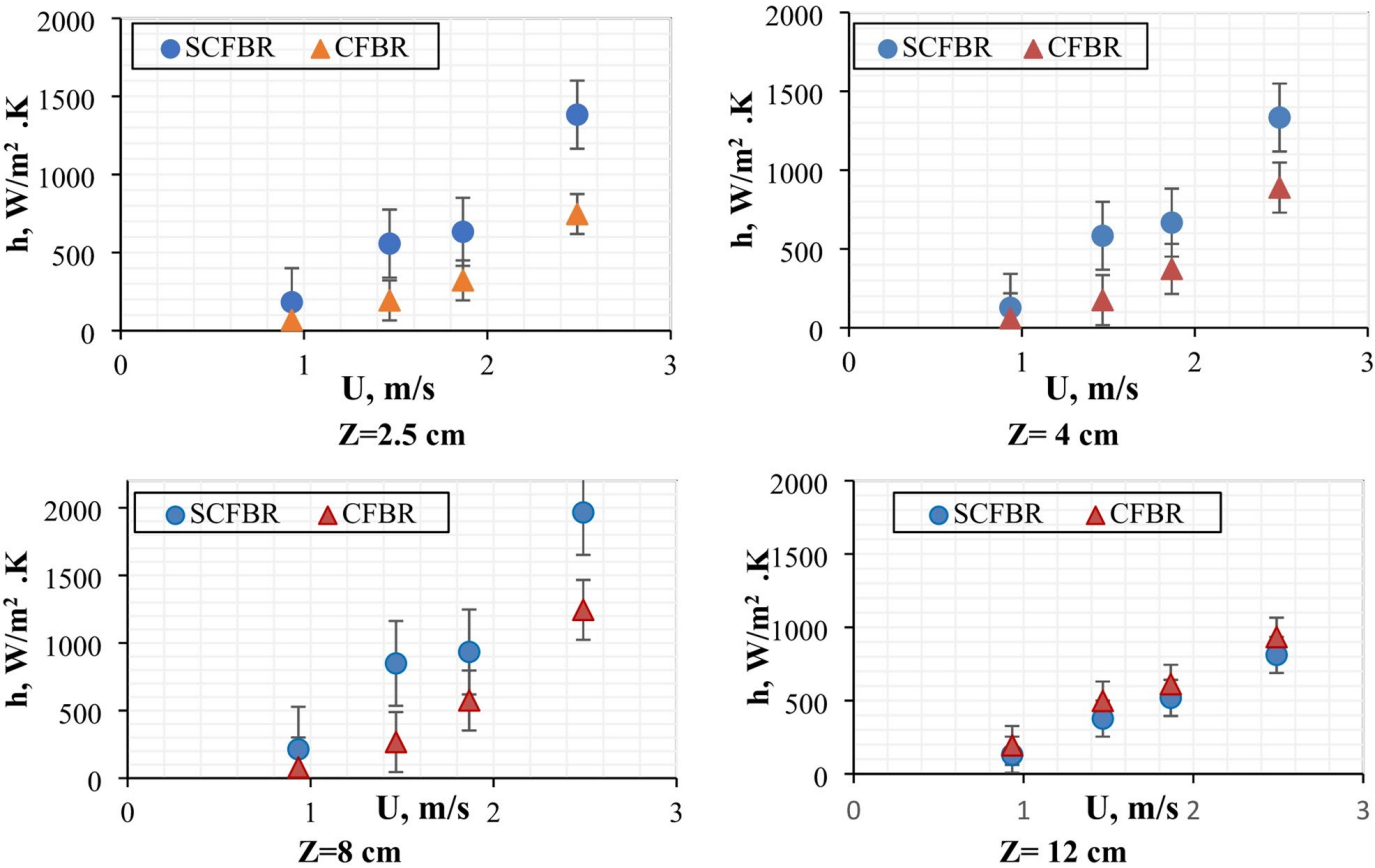
Radial distribution of heat transfer coefficient for CFBR and SCFBR at different inlet velocities, r/D = 0.5, and Z = 2.5, 4, 8, 12 cm.

Figure 9 shows the radial distribution of the heat transfer coefficient for CFBR and SCFBR at the center of the reactor (r/D = 0.5) at different inlet air velocities and different bed heights, Z of 2.5, 4, 8, and 12 cm. At a bed height of Z = 2.5 cm, the heat transfer coefficient of SCFBR was clearly higher than that of CFBR at all inlet velocities because of the swirling action that improves turbulence motion and particle-wall contact near the bottom of the reactor. The heat transfer coefficient continues to increase with the increase in bed height to 4 cm and 8 cm, emphasizing the sustained effect of the swirling flow across the height of the bed by decreasing temperature gradients, enhancing axial and radial mixing, and increasing thermal transport uniformity. At the highest bed height of 12 cm, the CFBR exhibited a slightly higher heat transfer coefficient than the SCFBR across all inlet velocities. This behavior may be attributed to the reduction in the swirling effect at higher bed heights due to the wall fraction and the collision of bed particles with the reactor wall, which dissipates energy, resulting in a decrease in the effect of swirling behavior in upper bed regimes. Furthermore, the increase in bed height improves the stabilization of flow in CFBR, enhancing the contact time while compensating for the absence of swirling action. These findings elucidate that the swirling behavior improves the heat transfer coefficient up to a certain height called the maximum swirling length, where the swirling action diminishes, leading to a decrease in the heat transfer coefficient. Therefore, the optimization of swirl energy and design is critical for different operating conditions and reactor scales.

**Fig. 10:**
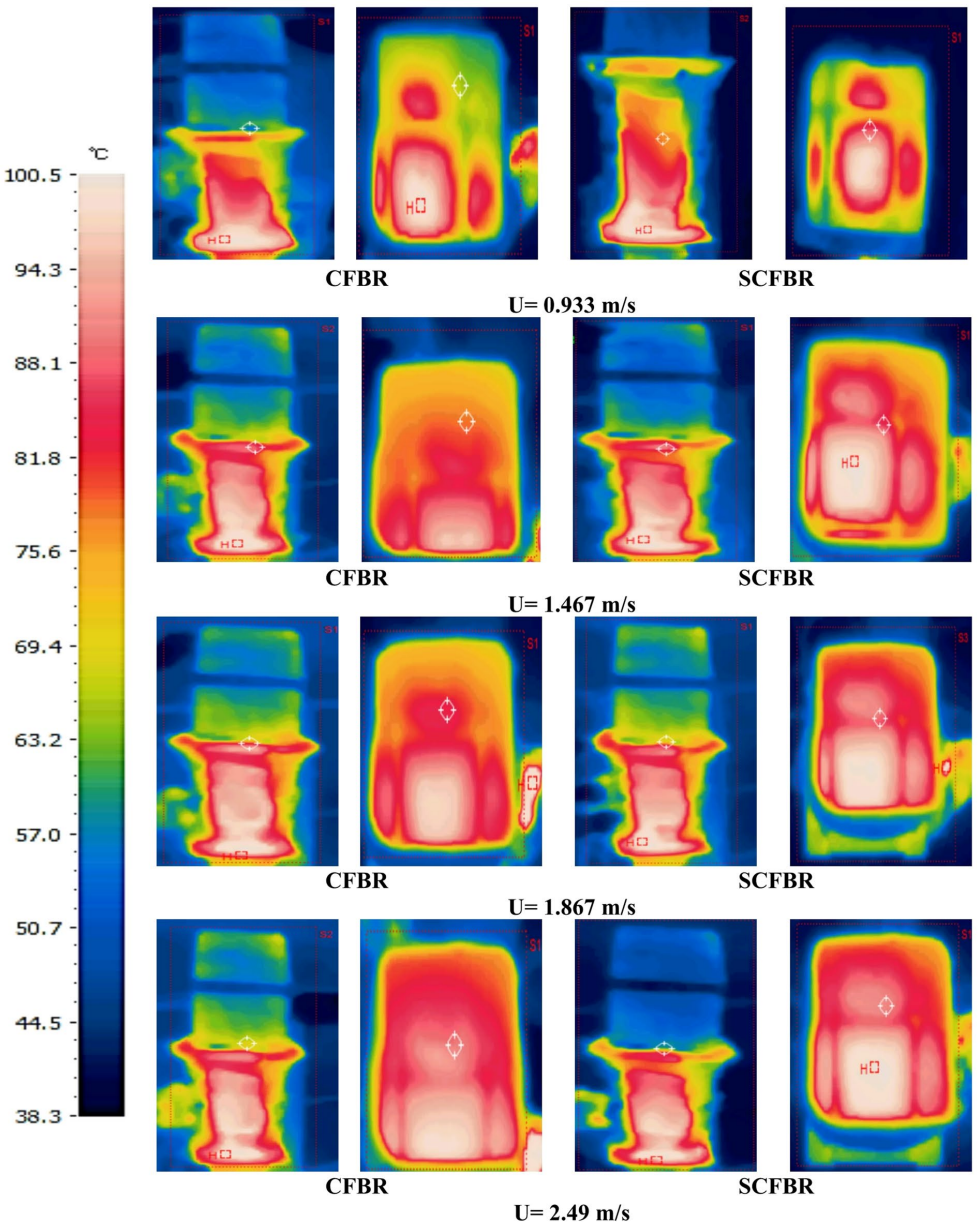
Photos of bed temperatures for CFBR and SCFBR at different inlet velocities measured at front and top views.

The surface particle temperature in CFBR and SCFBR was measured using an infrared camera at different inlet velocities. Figure 10 indicates the front and top views of particle surface temperature for CFBR and SCFBR. The front view images highlight a higher concentration of bed particles in the lower central bed region of CFBR, particularly evident in the lowest bed height. The lower region near the reactor inlet exhibited maximum thermal activity because of the direct contact of bed particles with inlet heated air and vigorous mixing among bed particles. With an increase in bed height, at the upper part of the reactor, the particle temperature rapidly decreased due to lower convective heat transfer and weaker gas-solid contact. The top image of surface particle temperature in CFBR indicates that the center region of the bed exhibited a relatively higher temperature than the periphery, suggesting that heat is initially stored in the core due to poor radial mixing. Furthermore, the outer regions of the bed show lower temperatures that may be attributed to heat loss through the reactor wall and lower gas-solid interaction. On the other hand, the front view of the surface particle temperature in the SCFBR shows a highly uniform temperature in the vertical direction. This is due to the swirling flow produced by the gas distributor, which enhances the vertical and lateral circulation, resulting in a higher homogenized thermal region. The region with high temperature in the SCFBR is extended compared to that of the CFBR, elucidating higher efficient heat transfer across the bed along with a decrease in thermal stratification. Additionally, the top view of the SCFBR indicates a more uniform temperature distribution, distinguished by circular hot regions extending outward from the bed center region. This considers the key result of the swirling flow, which increases radial mixing and improves the mobility of particles, allowing for more spread of heat through the bed area.

**Fig. 11:**
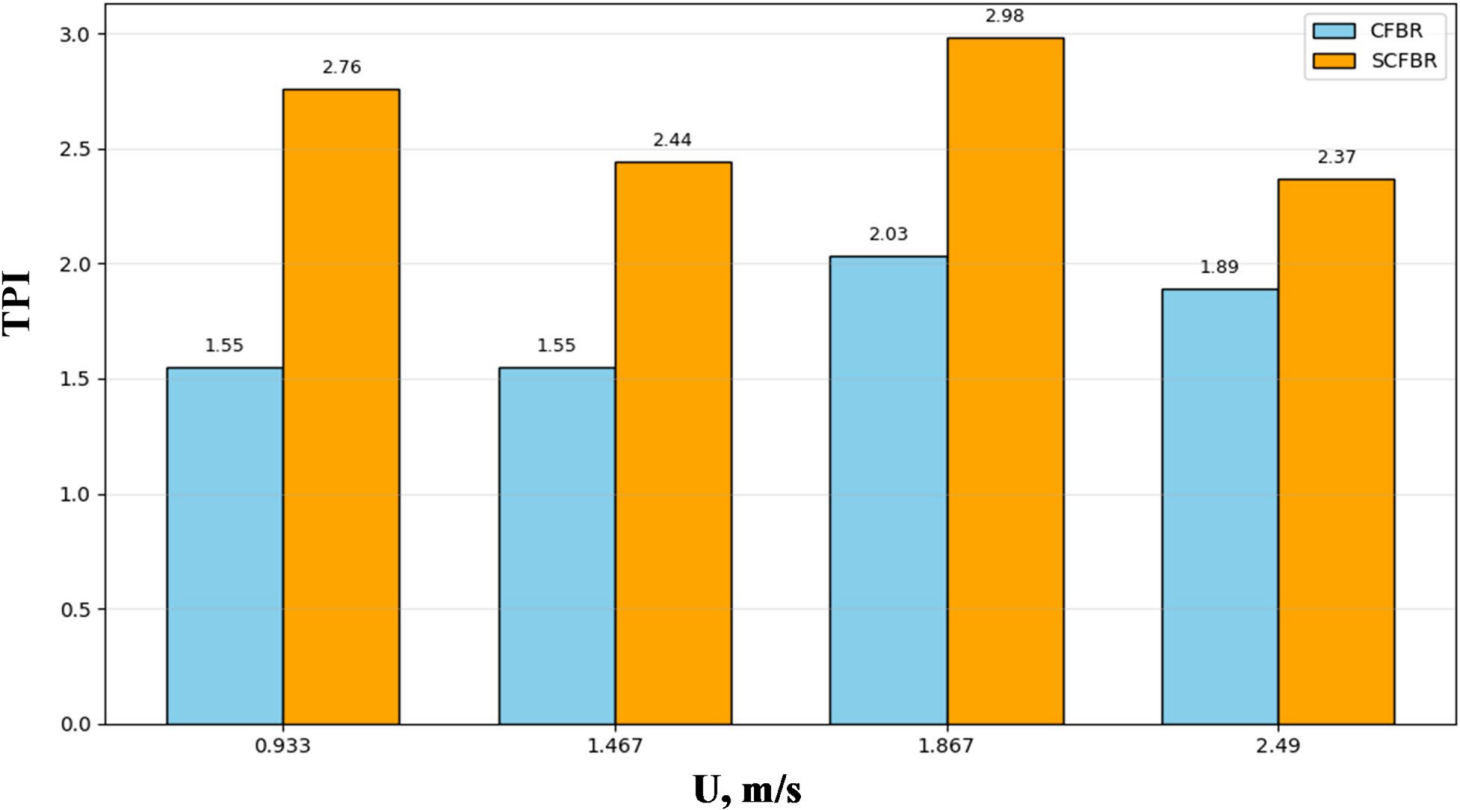
Comparison of thermal performance index between CFBR and SCFBR.

Figure 11 illustrates the comparison of the thermal performance index (TPI) for CFBR and SCFBR at different inlet velocities. The TPI is the ratio of the average heat transfer coefficient to its standard deviation, considered a strong metric for simultaneously estimating the heat transfer magnitude and its spatial uniformity. The results significantly emphasize that the TPI values of SCFBR were higher than those of CFBR, with the highest value of 2.98 at an inlet velocity of 1.867 m/s. This demonstrates that the blade distributor improved the average heat transfer coefficient in a more controlled and uniform manner. The lower change in the heat transfer coefficient for SCFBR is a direct advantage of strong homogeneous distribution among particles and intensive radial mixing due to minimizing localized dead zones and hot spots commonly observed in conventional FBRs. This coupling of high consistency and high performance is a major advantage for industrial applications, which require more stable thermal behavior to achieve product quality, process control, and longevity of the system.

### Machine learning results

**Fig. 12:**
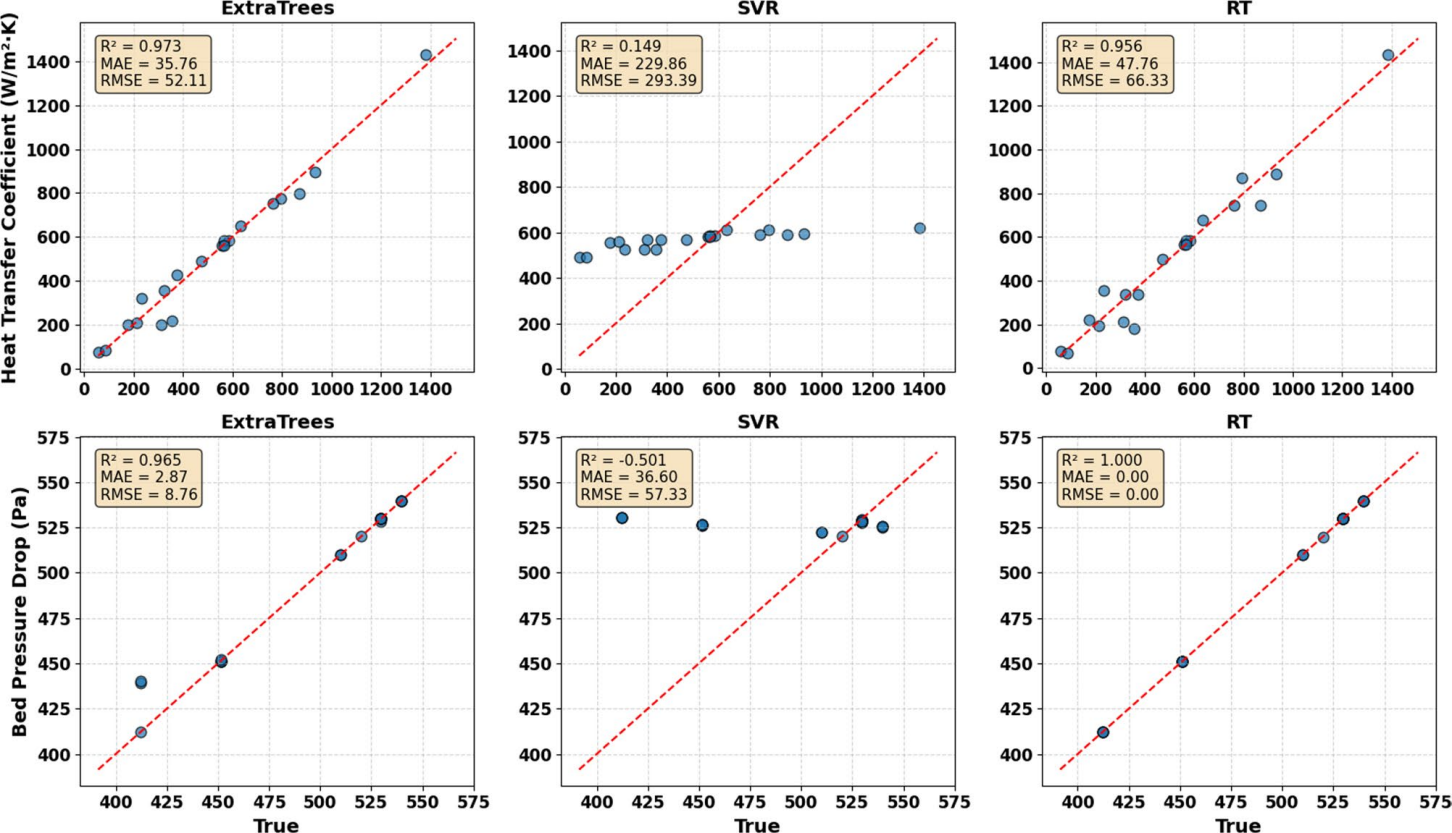
The scatter plots for different models.

Figure 12 illustrates a comprehensive comparison of the performance of three models in predicting the heat transfer coefficient and bed pressure drop. For the heat transfer coefficient, the scatter plot indicates that the ETR model achieved the highest accuracy, with an R² of 0.973, which means about 97.3% of the variance was explained in the experimental results, with an RMSE of 52.11 and a MAE of 35.76, demonstrating a relatively low mean error in this model. The SVR model exhibited lower performance, with a very high MAE of 229.86 and RMSE of 293.39 and a very low R² of 0.149, which reveals that the model failed to capture the complex relationships between the variables. The RT model also achieved higher accuracy, with a MAE of 47.76, RMSE of 66.33, and R² of 0.956, confirming its ability to predict performance​​. Regarding pressure drop, the scatter plot indicates an obvious superiority of the RT model with R² of 1.000, MAE and RMSE of 0.000. While the ETR indicated consistent and good performance with an R² of 0.965, RMSE of 8.76, and MAE of 2.87, making it the most reliable selection for pressure drop prediction. The SVR was also the weakest model, with a high RMSE of 57.33 and low R² of -0.504, reflecting its poor performance and unsuitability for modeling without parameter fine-tuning. While the RT model achieved a high value of R² of 1.000 and low values of MAE of 0.000 and RMSE of 0.000 for pressure drop prediction, such perfect metrics are unrealistic based on the experimental data, which reveals the overfitting of this model. With a limited dataset; the RT model easily memorizes the training data by achieving nodes that perfectly suit each observation, leading to perfect metrics. Moreover, the zero-training error is a case of overfitting that results from insufficient regularization constraints. This makes the ETR, which achieved more reliable and consistent performance, with R² of 0.965, RMSE of 8.76 Pa, and MAE of 2.27 Pa, the most suitable model for predicting pressure drop. Overall, the ETR model was a robust stochastic model based on clustering multiple trees; it has a high capability for capturing nonlinear data and noise, which illustrates its superior and balanced performance in both cases. These findings offer a scientific basis for selecting the optimal model in engineering applications, especially in the fields of energy and chemistry, where the efficiency of the reactor mainly relies on the accurate prediction of the heat transfer coefficient, which influences heating and cooling processes and pressure drop, which estimates the energy consumption. These results also demonstrate the effectiveness of the ETR as the best balance between accuracy and stability in predicting hydrodynamic and heat transfer FBR.

**Fig. 13:**
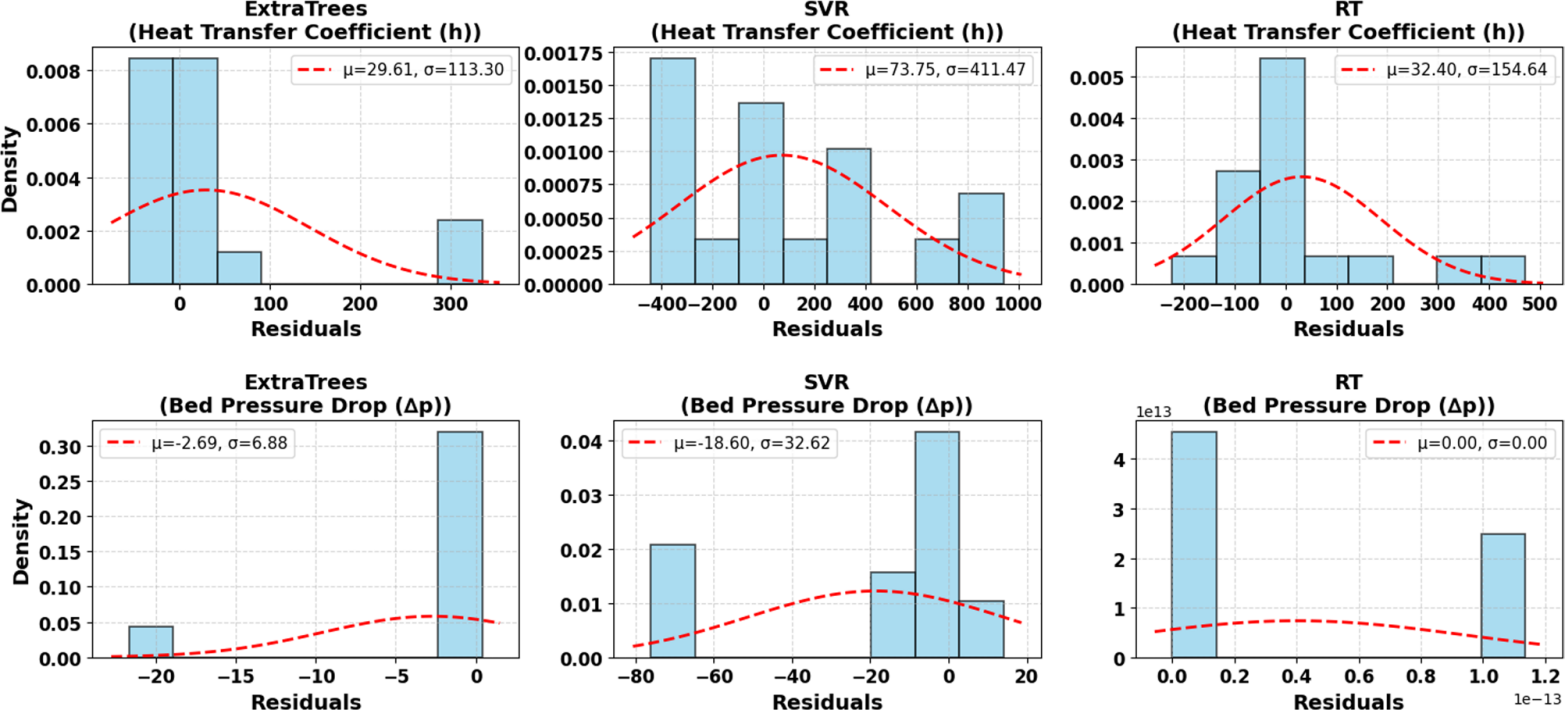
The residual histograms for heat transfer coefficient and bed pressure drop for different models.

The residual histograms for different models are presented in Fig. 13. These figures were achieved across a rigorous predictive modeling process using experimental results. After training the model, the residuals, which represent the difference between true or experimental data and predicted data, were determined for each target output h and ∆p. Then, the residual distribution histograms were plotted as a calibration to assess the accuracy and error profile of the model. As observed, ETR was the best in predicting h, with the lowest standard deviation of σ = 113.30 and a near-zero mean residual of µ = 29.61, reflecting its high predictive stability and accuracy. While the SVR model had a high negative residual of up to -800 and a very large deviation (σ = 411.47), which highlights that it may be improper for small and variable data or overestimated. Despite the simplicity of the RT model, it exhibited a good balance with σ of 154.64 and µ of 32.40, which makes it a good selection when transparency is more required than maximum accuracy. For the Δp, all models exhibited good performance, with very small residuals, as the standard deviations and near-zero were lower than 33 Pa. This demonstrates that the bed pressure drop primarily relies on air velocity, as confirmed by previous findings, making it a relatively easy factor to predict compared to heat transfer, which is affected by complex thermal variables. This finding is essential in the design of FBR because it illustrates that bed pressure drop can be accurately controlled by adjusting inlet velocity alone, while optimizing heat transfer requires more complex strategies involving control of bed and wall temperatures. Overall, these plots also confirm that ETR is the optimal selection for predicting heat transfer.

**Fig. 14:**
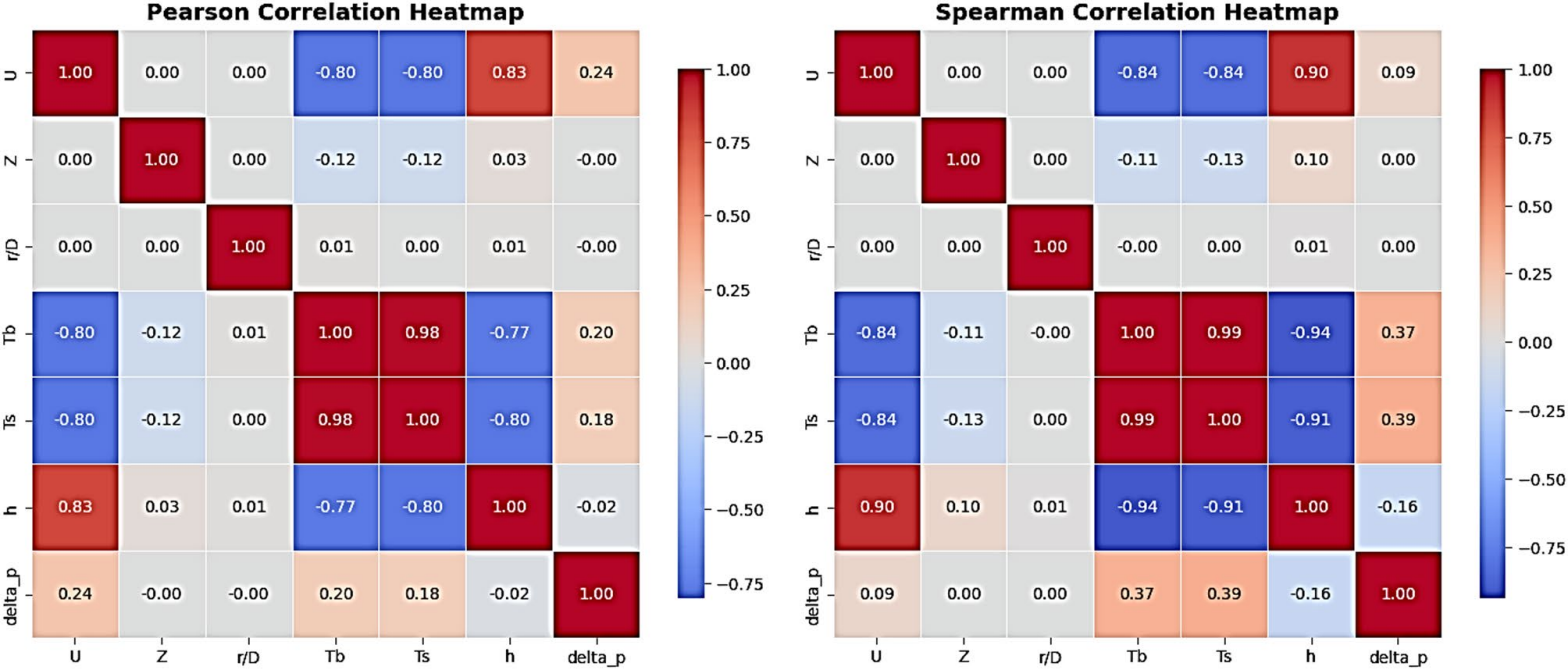
The pearson and spearman correlations heatmaps.

Figure 14 displays the results of Pearson and Spearman correlation analyses, which were used to explain relationships among parameters. For h, the inlet velocity (U) had the strongest relationship, with Pearson’s coefficient of 0.83, which illustrates that the increase in air velocity directly enhances heat transfer. This is theoretically expected due to improved mixing of bed particles and a decreased stable shell layer around the reactor wall. The analysis also indicates strong negative relationships with bed temperature (Tb) and wall temperature (Ts), with coefficients of -0.77 and − 0.80, respectively, demonstrating that heat transfer is highest with the increase in the temperature difference between the wall and the bed because h increases with a decrease in bed and wall temperatures at constant U, which agrees with Newton’s law of heat transfer (q = h·ΔT). On the other hand, both Z and r/D show no meaningful correlation with h, as their values ​​are close to zero. This demonstrates that the heat transfer distribution in the bed is not a strong function of axial or radial position in this experimental range and can be considered relatively uniform throughout the bed height or distance from the center. Regarding the pressure difference (ΔP), the correlations are very weak with all variables, with 0.24 for U, 0.19 for T_b_, and 0.18 for Ts. This indicates that ΔP is not highly sensitive to the variation in these parameters based on the obtained experimental results, or that it depends on other parameters such as reactor geometry or particle density and size. Additionally, the similar values of the Pearson and Spearman coefficients indicate that the relationships are nearly linear, with no significant effect from abnormal distributions. However, the slight difference in some pairs, such as h vs. Tb: -0.77 vs. -0.94, illustrates a more monotonic relationship than a linear one, where the variation direction is more important than the precise linear magnitude. Overall, this analysis elucidates that the inlet air velocity is the most powerful variable in enhancing heat transfer, while the pressure differential remains relatively stable under different operating conditions.

**Fig. 15:**
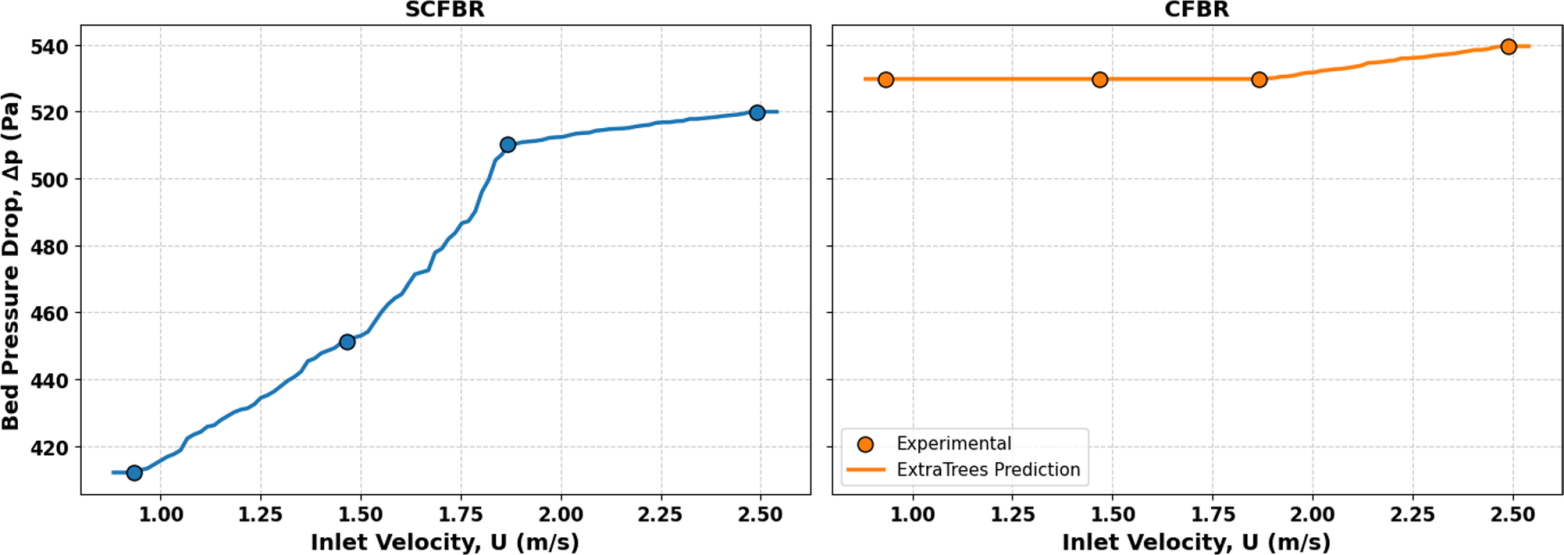
The experimental and predicted pressure drop for SCFBR and CFBR.

The measured and predicted bed pressure drop for CFBR and SCFBR is shown in Fig. 15. The figure highlights a high degree of agreement between the experimental and the predicted results for both designs, which confirms the accuracy of the ERM used and its capacity for reliably capturing the hydrodynamic behavior of the FBR. The pressure drop increases with an increase in inlet air velocity for both reactors, which is a physically expected trend because velocity increases drag forces and interaction between the gas and solid phases within the bed. On the other hand, the figure significantly illustrates that the pressure drop ​​in the SCFBR is lower than that in the CFBR at all inlet velocities. The reduction in bed pressure drop is attributed to the swirling flow impact, which promotes the distribution of solid particles and decreases the agglomeration and channeling within the bed, leading to a decrease in the overall flow resistance. Additionally, the swirling motion facilitates smoother movement of particles and improves the radial mixing, directly decreasing the energy required to generate the fluidized state. The high observed degree of convergence between the experimental and predicted results for two reactors reveals that the predictive model successfully captures the impacts of conical geometry and swirling flow on pressure drop with good accuracy.

**Fig. 16:**
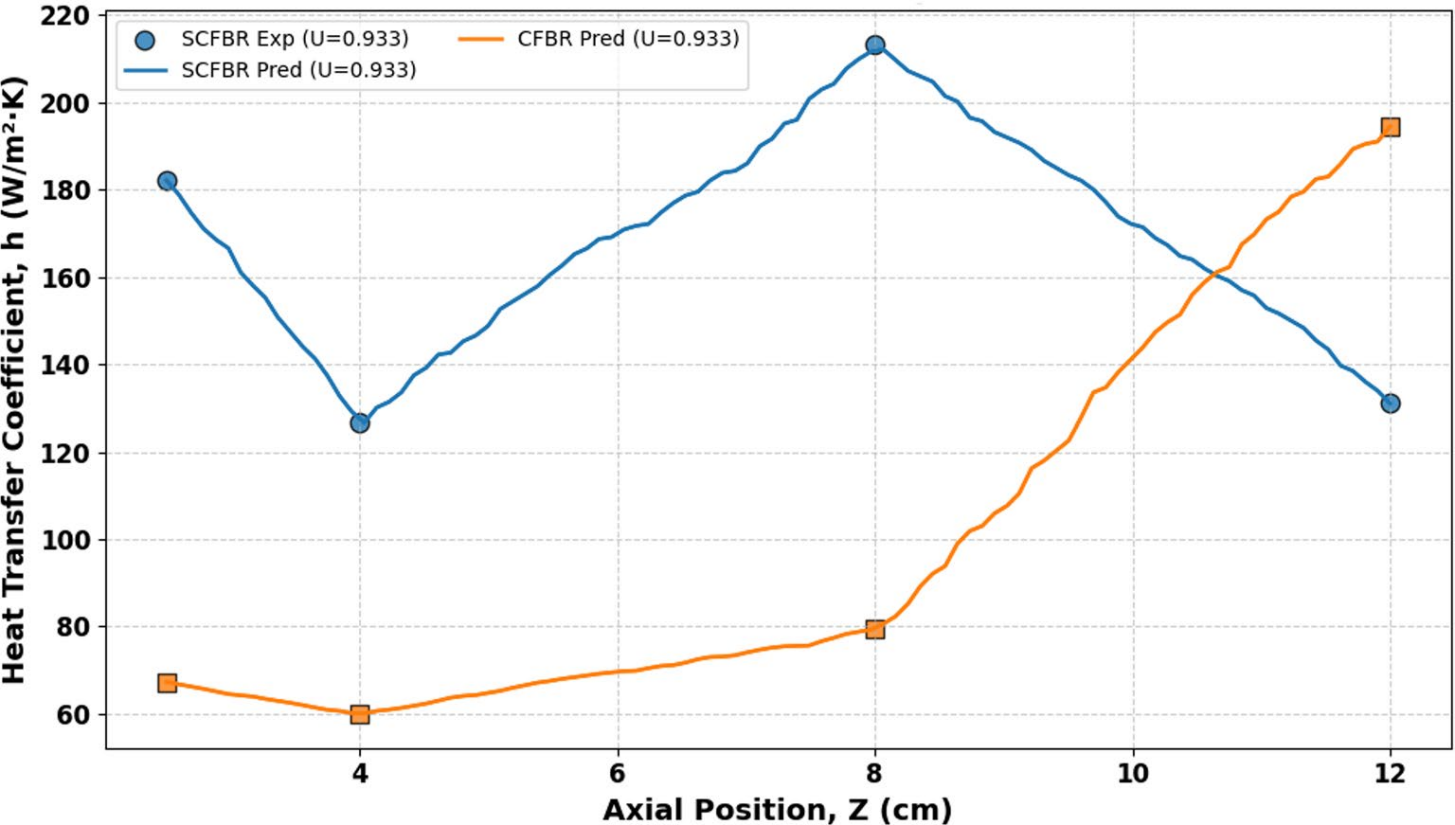
The experimental and predicted axial distribution of heat transfer coefficient for SCFBR and CFBR.

**Fig. 17:**
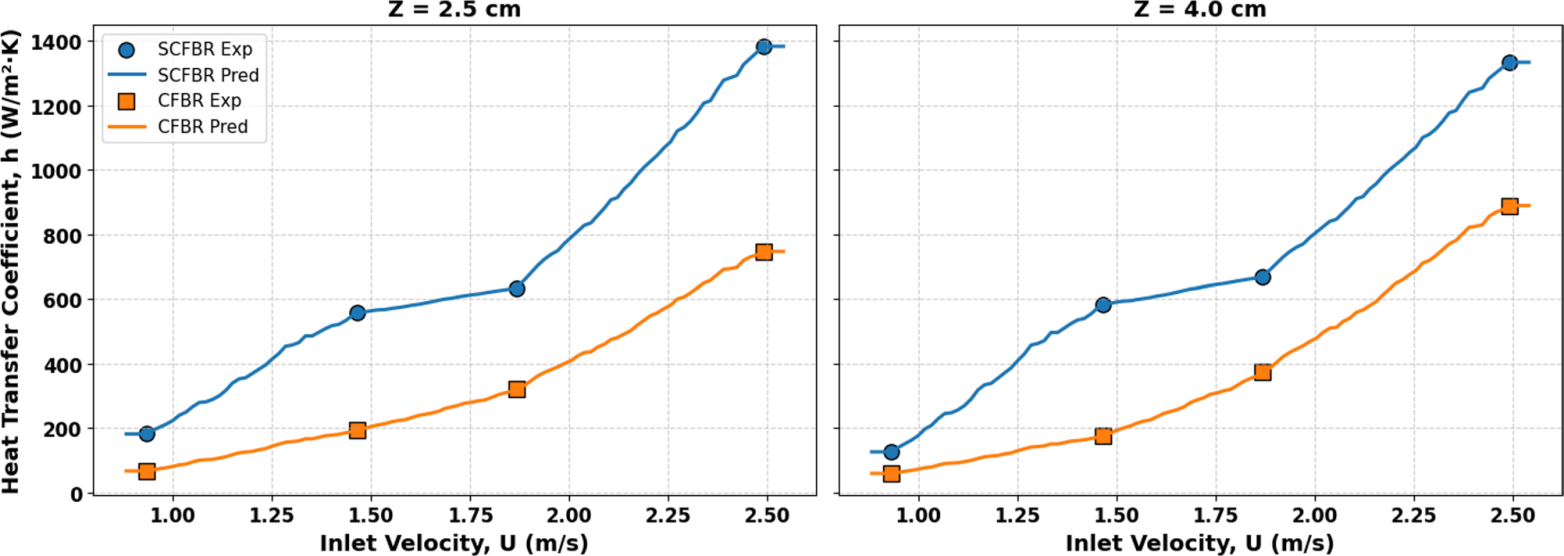
The experimental and predicted radial distribution of heat transfer coefficient for SCFBR and CFBR.

Figures 16 and 17 illustrate a comparison between the experimental and predicted values ​​of the axial (at r/D of 0.5) and radial distribution of the heat transfer coefficient for CFBR and SCFBR, respectively. Generally, in both reactors, the heat transfer coefficient increased ​​in the lower zones and then gradually reduced with an increase in axial height. This is a physically predicted behavior due to the reduction in the density of particles and in contact intensity between the bed particles and the reactor wall as they move away from the air inlet. In the SCFBR, the heat transfer coefficient was higher than that in the CFBR at most heights, especially in the lower and middle regions. This is due to the impact of swirling flow, which promotes axial and radial mixing and enhances the rate of hot particles hitting the reactor wall, leading to an obvious enhancement in heat transfer. In contrast, the axial heat transfer coefficient in the CFBR shows a lower uniform distribution, with heat transfer concentrated near the center region because of the dominance of axial flow and poor radial mixing, which decrease the efficiency of heat transfer at the wall. Figure 17 illustrates that the heat transfer coefficient varies with radial location, with higher values near walls and lower values in the center regions. This is a consequence of the increased frequency of particle collisions with the reactor wall and enhanced conduction and convection mechanisms in the near-wall zones. This behavior is most observed in the SCFBR, which shows higher heat transfer coefficient values ​​at most radial locations. This increase in the SCFBR is attributed to the swirling flow impact, which produces additional tangential and radial velocity components. This improves the movement of particles from the center region towards the wall, enhances the distribution uniformity, and hence intensifies the thermal interaction between the particles and the wall. Figures 16 and 17 also elucidate good agreement between the experimental and predicted results for both reactors, reflecting the capability of the model for accurately representing the heat transfer in both radial and axial directions.

The higher accuracy of the ML models, particularly ETR, as indicated by the high values of R^2^ and low values of RMSE, as observed in the previous figures (15–17), is a consequence of capturing the nature of the hydrodynamic and heat transfer processes within the SCFBR. The experimental measurements of this study differ from other stochastic datasets, as they exhibit consistent physical behavior with lower noise. Moreover, the use of ensemble-based algorithms like ET and RF allows for the effective capture of non-linear interactions between operational parameters while decreasing variance across the averaging of multiple decision trees. This synergy between robust algorithmic architecture and high-quality experimental results leads to a predictive model that accurately captures the physical reality of the FBR.

**Fig. 18:**
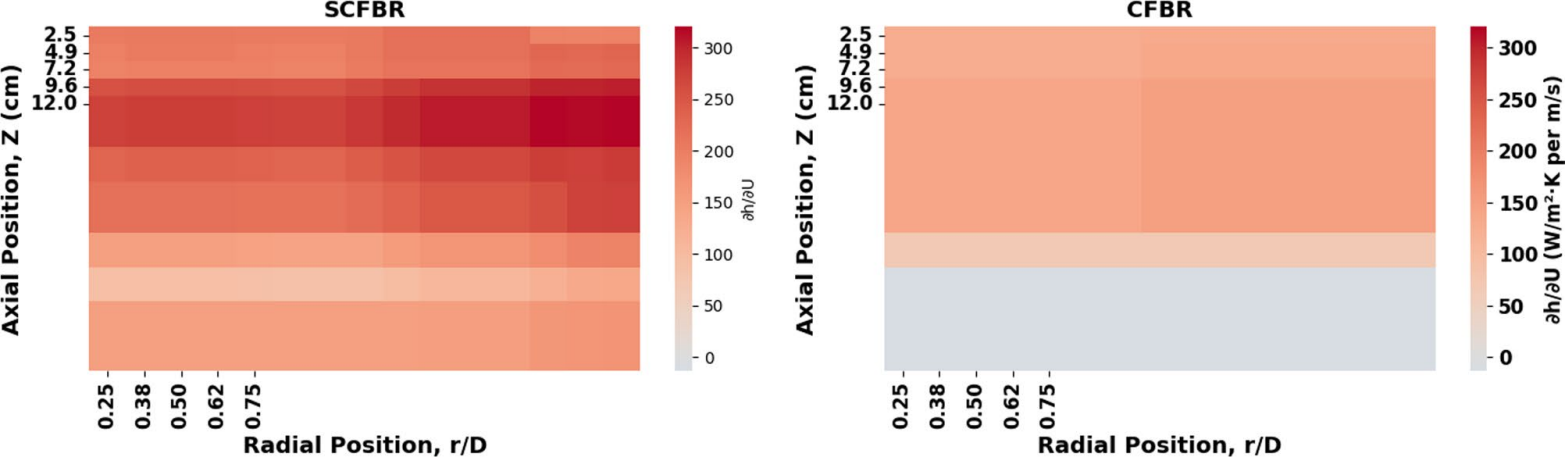
The local sensitivity analysis for SCFBR and CFBR.

Figure 18 presents a thermal map of the local sensitivity of the heat transfer coefficient to the inlet velocity (∂h/∂U) at a constant velocity of 1.8 m/s, distributed along the axial (Z) and radial (r/D) directions for CFBR and SCFBR. This map was developed using accurate numerical analysis with the ETR model. The partial derivative ∂h/∂U was estimated using the central difference approximation: ∂h/∂U ≈ [h (U + δ) – h (U − δ)] / (2δ), where δ = 0.01 m/s, to decrease the approximation error and achieve high accuracy. In the SCFBR, the map highlights a high heat (red) in the mid-height (Z = 4–8 cm) at the central region (r/D = 0.5), which elucidates the presence of a swirling core where particle mixing is strong. The increase in the inlet velocity results in a clear increase in heat transfer, which illustrates the high value of ∂h/∂U (~ 300 W/m²·K per m/s). This agrees with the previous finding that the heat transfer coefficient increases sharply with velocity in the SCFBR. The sensitivity of the CFBR appears lower and more consistent at all locations, illustrating that the conical geometry homogenizes the flow and heat. This makes the reactor less sensitive to local variations in velocity and thus more predictable but less responsive to optimization. The lower sensitivity at the bed top at Z of 12 cm and near the walls at r/D of 0.25 and 0.75, respectively, is also physically justified. This is because the velocity effect minimizes near the walls due to the boundary layers and reduced rotation; while at the top, the lower particle concentration and heat loss reduce the bed temperature, which decreases the driving force (ΔT) of heat transfer and hence the marginal advantage of increasing velocity. Therefore, this heat map is a proven geometric statistic, demonstrating that controlling the operation within the swirling core of the SCFBR at mid-height can maximize the energy return on investment. For the CFBR, any location can be considered with equal confidence as the sensitivity is uniform. This type of analysis that integrates physical interpretation with ML, is rarely achievable experimentally, illustrating the potential of using ML approaches to help in the design of next-generation reactors.

### Statistical analysis

**Fig. 19:**
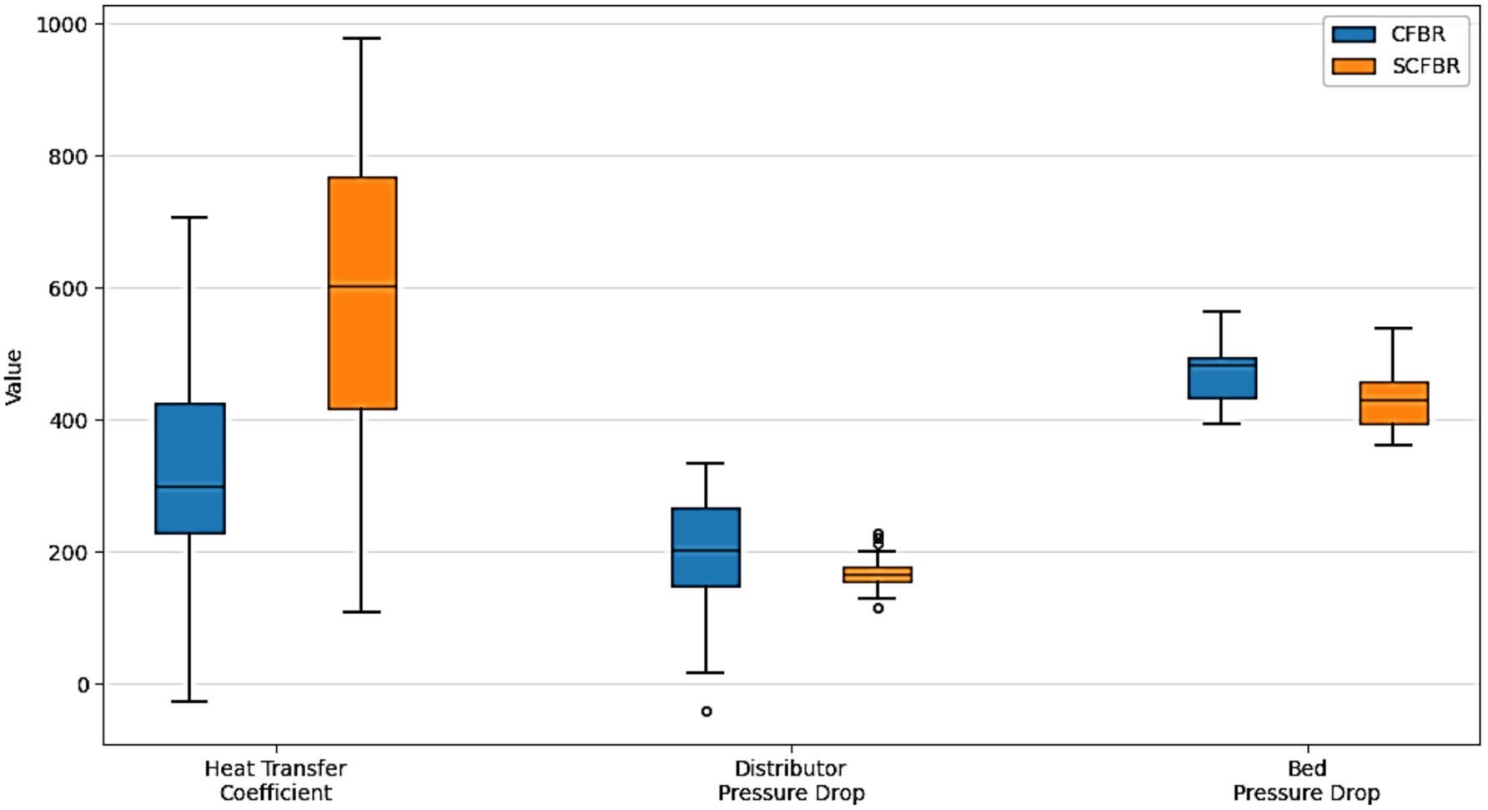
Comparison of distributor pressure drop, bed pressure drop, and heat transfer coefficient between CFBR and SCFBR.

In this study, a one-way analysis of variance (ANOVA) and Welch’s t-test were applied to statistically estimate the effect of distributor configuration on pressure drop and heat transfer coefficient for conventional CFBR and SCFBR. The pressure drop analysis shows that both the distributor and bed pressure drop of SCFBR are lower than those of CFBR at all inlet velocities, confirming that the blade distributor introduced more uniform and efficient gas distribution with lower localized resistance. Moreover, swirling flow can minimize channeling and create a more loosely packed, homogeneous particle distribution, which decreases the overall resistance of flow. The t-test on the bed pressure drop had a p-value of 0.0008 and a t-statistic of 4.21, showing a statistically remarkable difference, with *p* < 0.05, between SCFBR and CFBR, demonstrating the significant enhancement of bed hydrodynamics in SCFBR. For the heat transfer process, the statistical analysis shows a substantial enhancement in SCFBR due to increasing particle-wall contact and higher radial mixing. This enhancement is confirmed by ANOVA analysis, which achieves a p-value lower than 0.0001 and an F-statistic of 34.7. This was further validated by Welch’s t-test, yielding a p-value lower than 0.0001 and a t-statistic of 5.89. The large effect size, Cohen’s d = 1.47, and significant p-value suggest a strong statistical proof that a blade distributor achieves a reliable and dramatic enhancement in the hydrodynamics and thermal performance of the reactor. Figure 19 shows an accurate and clear visual comparison of the flow behavior and heat transfer between CFBR and SCFBR based on statistical analysis results.

**Fig. 20:**
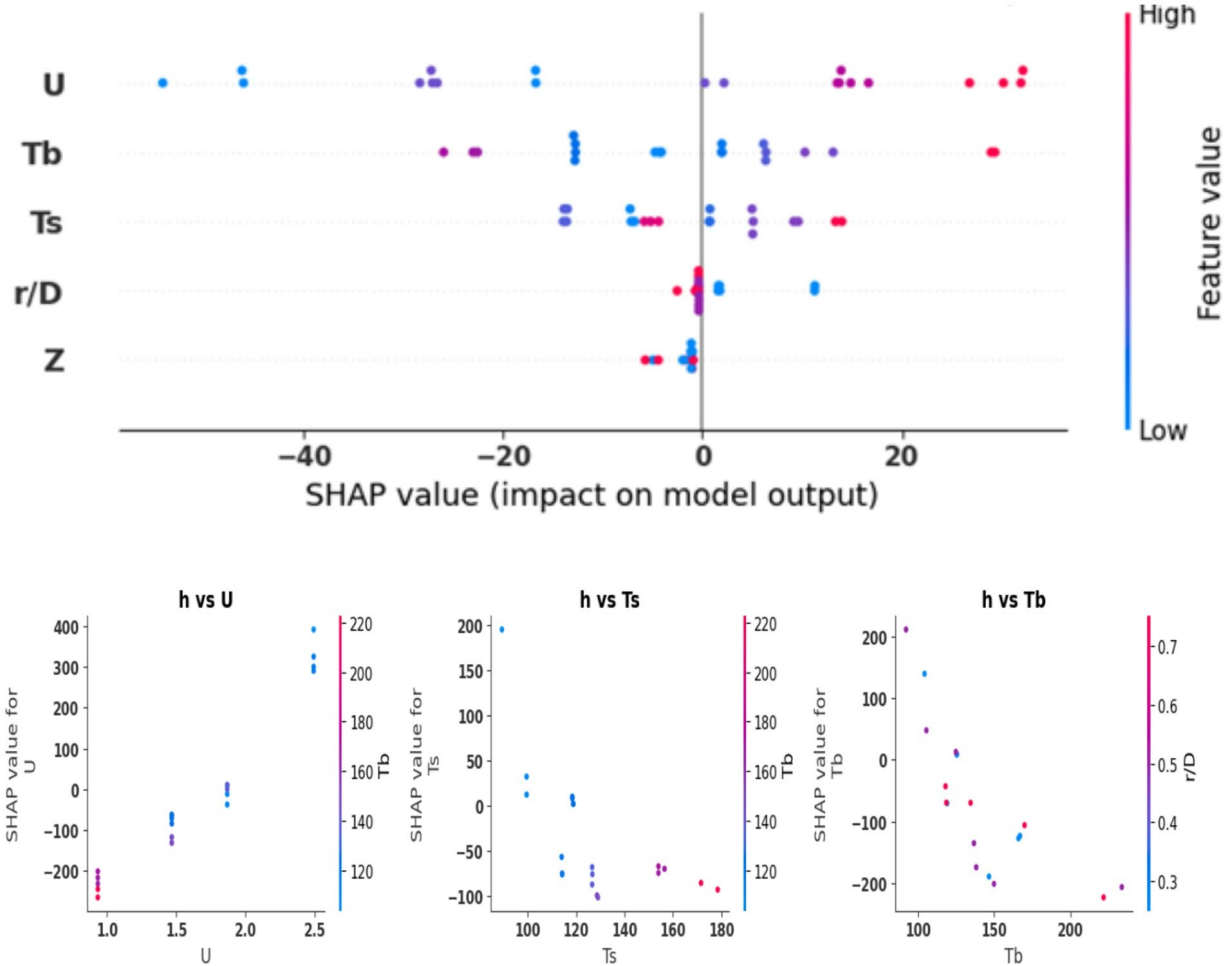
SHAP analysis for heat transfer coefficent.

**Fig. 21:**
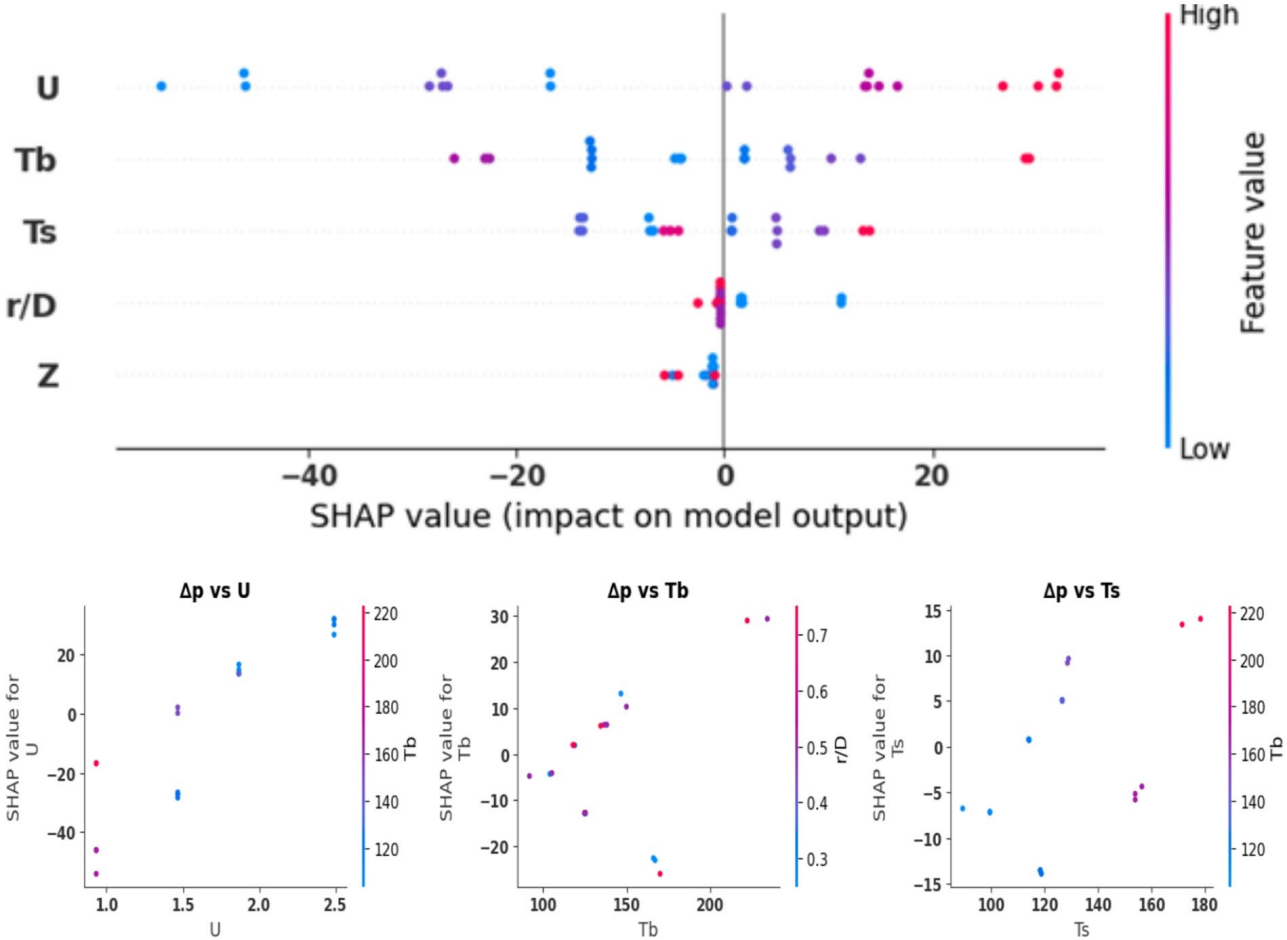
SHAP analysis for bed pressure drop.

Figures 20 and 21 present the results of the SHAP analysis for heat transfer (h) and pressure drop (Δp), respectively. The analysis shows a broad range throughout all inputs and offers more detailed interpretations compared to Pearson and Spearman’s analyses, which are restricted to illustrating the direction and strength of the linear or ordinal relationship between the variables. Regarding the heat transfer coefficient, the SHAP analysis reveals that the inlet air velocity is the most effective parameter, as the increase in velocity leads to significant improvements in radial and axial mixing and increases the frequency of particle-wall collisions that improve the heat transfer coefficient. The analysis also highlights that the impact of velocity is nonlinear and relies on the interaction with other variables, which can’t be accurately captured by Pearson and Spearman analyses. The SHAP analysis also indicates that both bed and wall temperatures have a negative impact on h; however, this influence changes based on the inlet velocity and other operating conditions. With an increase in bed or wall temperature, the temperature difference driving heat transfer declines, which decreases heat transfer coefficient. Although this is a physical agreement with Newton’s law of heat transfer, it appears in SHAP analysis as a localized quantitative relationship describing the contribution of each variable under different operating conditions, instead of as a fixed general relationship as in correlational analyses. For axial height and the radial position, SHAP analysis emphasizes their limited effect on the heat transfer coefficient compared to velocity and temperature. However, they play a minor role by enhancing particle distribution and mixing intensity, particularly in certain bed regions. This impact was not clearly handled in Pearson and Spearman due to its overall linear weakness. For the pressure drop, SHAP analysis confirms that the inlet velocity is the dominant variable, as the rise in it directly results in an increase in bed pressure drop because of increased flow resistance and drag within the bed. While other parameters, including temperature and axial and radial positions, exhibit minor and lower impacts on bed pressure drop, this explains the results of Pearson and Spearman analyses, which indicated weak correlations with these factors. However, SHAP analysis goes beyond this general characterization by illustrating that these factors may have an indirect or lower influence under certain operating conditions, but this effect remains less significant than the that of inlet velocity. Compared to Pearson and Spearman analyses, SHAP analysis offers a comprehensive physical-statistical insight that illustrates the contribution of each individual factor across diverse operating conditions, which makes it a more suitable option to describe heat transfer and pressure drop behavior within the FBR, and to support design and operational optimization decisions based on ML model.

## Conclusion

This study presents a comprehensive investigation of bed hydrodynamics and the heat transfer process of a novel swirling conical fluidized bed reactor through experimental analysis and ML modeling. Although both conical fluidized bed and swirling fluidized bed reactors have well-established advantages, few studies have addressed their combined impacts. The study’s novelty lies in using the blade distributor in the conventional conical fluidized bed, employing infrared thermography for direct and non-intrusive measurement of particle surface temperature, and utilizing ML techniques to capture the relationships within the reactor. The main results of this study confirm that using a blade distributor in a traditional conical fluidized bed improves its performance as follows:

- Both the distributor and pressure drop of SCFBR decreased compared to those of CFBR, which reduces the required input energy.
- The thermal performance of SCFBR was improved by increasing the bed-to-wall heat transfer coefficient by up to 40% higher than that of CFBR.
- The infrared thermography shows more temperature uniformity and a thermal field in SCFBR, especially in the lower and middle regions of the bed.
- The effect of swirling flow diminishes at higher bed locations, indicating a critical swirling height beyond which energy losses surpass mixing advantages.
- The extra tree model exhibited the highest accuracy in predicting the heat transfer coefficient, with an R² of 0.973, an RMSE of 52.11, and a MAE of 35.76, as well as bed pressure drop with an R² of 0.965, an RMSE of 8.76, and a MAE of 2.27.
- SHAP analysis emphasized that inlet air velocity is the dominant variable in both heat transfer and pressure drop, with secondary but influential roles for temperature and axial and radial positions, which offer deep insight that intensifies the predictive models’ results.

## Limitations and future work

Whilst this study provides promising results that emphasize the advantages of the SCFBR across an integration of experimental and ML modeling, some limitations remain. The study used a laboratory-scale reactor with fixed dimensions without considering the impact of scale-up. Future work should investigate the impacts of larger scales and the variation of cone angles to establish general design correlations. Using sand as bed material, while industrial applications usually use a mixture of complex particles, representing a limitation. Therefore, a mixture with different irregular shapes, sizes, and thermal properties should be used in future investigations. Despite the developed ML models achieving high accuracy, their generalizability to different particle materials and reactor shapes needs further validation. Future work is recommended to use 3D-CFD simulations to capture the swirling flow characteristics in more detail, which would complement the experimental findings.

### Ethics approval

The authors declare that the submitted manuscript is original. They acknowledge the current review has been conducted ethically, and the final shape of the research has been agreed upon by all authors.

## Data Availability

The data that support the results of this study are not openly available due institutional and intellectual-property restrictions associated with the research implementation and its future extension.
